# Learning relative values in the striatum induces violations of normative decision making

**DOI:** 10.1038/ncomms16033

**Published:** 2017-06-20

**Authors:** Tilmann A. Klein, Markus Ullsperger, Gerhard Jocham

**Affiliations:** 1 Cognitive Neuroscience, Center for Behavioral Brain Sciences, Otto-von-Guericke-Universität Magdeburg, Universitätsplatz 2, 39106 Magdeburg, Germany; 2 Institute of Psychology, Otto-von-Guericke-Universität Magdeburg, Universitätsplatz 2, 39106 Magdeburg, Germany; 3 Center for Behavioral Brain Sciences, Otto-von-Guericke-Universität Magdeburg, Universitätsplatz 2, 39106 Magdeburg, Germany; 4 Max Planck Institute for Human Cognitive and Brain Sciences, Stephanstraße 1a, 04103 Leipzig, Germany; 5 Day Clinic for Cognitive Neurology, University Hospital Leipzig, Liebigstraße 16, 04103 Leipzig, Germany

## Abstract

To decide optimally between available options, organisms need to learn the values associated with these options. Reinforcement learning models offer a powerful explanation of how these values are learnt from experience. However, human choices often violate normative principles. We suggest that seemingly counterintuitive decisions may arise as a natural consequence of the learning mechanisms deployed by humans. Here, using fMRI and a novel behavioural task, we show that, when suddenly switched to novel choice contexts, participants’ choices are incongruent with values learnt by standard learning algorithms. Instead, behaviour is compatible with the decisions of an agent learning how good an option is relative to an option with which it had previously been paired. Striatal activity exhibits the characteristics of a prediction error used to update such relative option values. Our data suggest that choices can be biased by a tendency to learn option values with reference to the available alternatives.

## Abstract (web summary)

Though people learn that certain choices may be more advantageous, they often do not choose this option. Here, the authors explain this behaviour: people learn how good a choice is relative to the choices it has been associated with previously, and this learning takes place in the striatum.

Adaptive, goal-directed behaviour requires an individual to estimate the reward that can be expected from a particular stimulus or action. According to reinforcement learning theories, such value estimates are learnt through prediction errors: the difference between expected reward and reward actually obtained^1^. These models have become very influential in neuroscience, and we now know that the firing of midbrain dopamine neurons during learning reflects the prediction error from a class of algorithms known as temporal difference learning^2^. This dopaminergic prediction error is thought to be used as a teaching signal by modulating synaptic plasticity in target areas, in particular, in the striatum^3^,^4^. Accordingly, many human neuroimaging studies have found prediction errors represented by striatal activity^5^,^6^,^7^,^8^,^9^,^10^. The exact nature of prediction errors encoded in the brain, however, has been less clear. This raises the more fundamental question of the underlying computational mechanisms that govern behaviour. The reinforcement learning literature offers a number of algorithms for learning the value of particular stimuli or actions, or for directly updating a stochastic choice rule without learning any value estimates at all^1^. Here, we suggest that humans do indeed learn option values, but that these values are learnt with reference to the currently available alternatives. We further suggest that this relative value learning can systematically give rise to suboptimal decision-making behaviour in new contexts.

We recently showed that when participants were allowed to reject an option offered to them and observe the gain or loss they would have incurred for choosing that option, striatal activity on these reject trials represented a prediction error with reversed sign. We could further show that this was not merely a regret signal^10^. This led us to hypothesize that values are learnt relative to the available alternatives: experiencing an outcome of zero after a reject decision should increase the value of rejecting upon observing that accepting the offer would have resulted in a loss. Such referencing of the available alternatives has already been shown for outcome-related activity in the striatum, when outcomes were drawn from a lottery^11^. In an environment where past experiences can be used to predict future outcomes, it might be adaptive that learning also occurs in such a relative frame of reference. In humans, such relative learning can account for the finding that learning to avoid an aversive stimulus is as efficient as learning to approach an appetitive stimulus, despite the absence of continued negative reinforcement from the avoided stimulus^12^. Thus, relative learning can explain symmetrical performance where previous computational accounts would have predicted asymmetrical performance. Here, we asked whether relative value learning in the appetitive domain may explain suboptimal choice behaviour where previous normative accounts would predict rational behaviour.

A person who generally eats in mediocre restaurants that only offer poor to medium quality wine may, when dining in a novel restaurant, prefer the familiar, medium quality wine over a higher quality wine. The decision maker would be biased if they had usually experienced the mediocre wine together with very poor wines, and the top-quality wine together with other very good ones. This suggests the intriguing possibility that decisions that appear to violate transitivity may actually arise naturally from such a relative value learning mechanism.

We designed a novel reinforcement learning task in which absolute and relative value learning algorithms make opposite predictions about choice behaviour upon transition to novel contexts. We find that behaviour is indeed consistent with participants learning relative option values. In new contexts, participants show a systematic bias towards options that had previously been paired with lower-value options, even when those have an objectively lower reward value. Signals in the striatum display the full characteristics of a relative value prediction error. In contrast, we find no signals in the striatum or elsewhere in the brain that could be used for updating the objective values of options.

## Results

### Behavioural task

**Figure 1:**
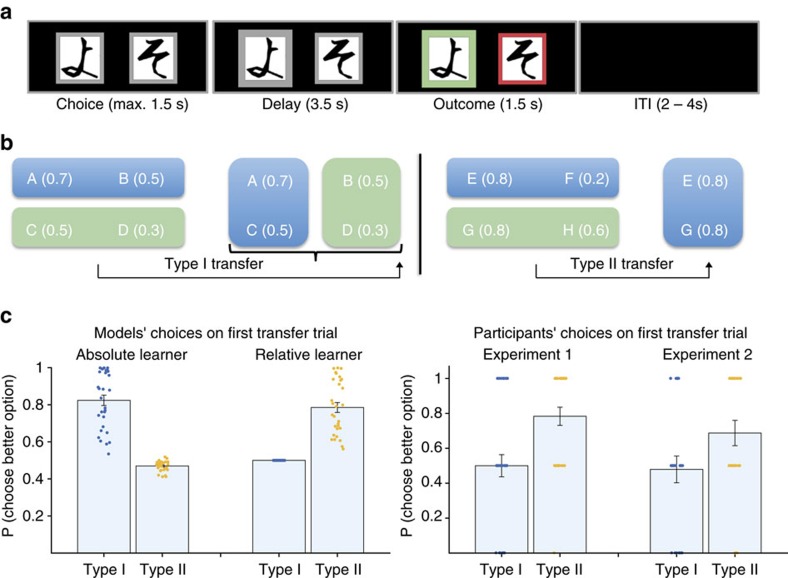
Task structure and model predictions and behaviour. (**a**) Schematic of the experiment. On each trial, two abstract symbols were presented that were randomly assigned to a reward probability that remained fixed, but was independent between the two options. Subjects could select the options by pressing a button with the index finger of the right or left hand, respectively. Side of option presentation was randomized. After a delay, the outcome (reward or no reward) was revealed for both the chosen and unchosen option (colour of the frame turning green or red). (**b**) After 30 acquisition trials, subjects were suddenly exposed to novel combinations, which we refer to as TYPE I or TYPE II transitions. In TYPE I transitions, the novel combinations were such that in each pair, there was always one option that had acquired a higher absolute value (A in AC and B in BD), but by virtue of its previous pairing had acquired the same relative value. By contrast, in TYPE II transitions, there were always two options that had acquired an identical absolute value (E and G, performed twice with different symbols), but by virtue of previous pairing, E had acquired a higher relative value. (**c**) Model and real subject behaviour. Values are mean±s.e.m. probability across subjects to select the better option (A or B) on TYPE I transitions, or option E (higher relative value) on TYPE II transitions. Reinforcement learning models learning either absolute or relative option values were fit to subjects’ behaviour during acquisition (left). The value estimates and softmax choice temperature were used to generate model choice probabilities for the first transfer trial, before any new learning could occur. The two classes of models make exactly opposite predictions about behaviour on TYPE I and TYPE II transitions. In both Experiments 1 and 2, subjects’ behaviour is consistent with relative value learning: Participants are indifferent on the first trial of TYPE I transitions, but show a systematic preference on TYPE II transitions.

Participants saw two pairs of stimuli, AB and CD (in random sequence), that were probabilistically associated with reward (0.7 versus 0.5 for AB, 0.5 versus 0.3 for CD). Participants’ task was to learn by trial and error to select the better option. After 30 trials of this learning stage, participants were suddenly switched to a transfer stage, where they now had to make choices between A versus C and B versus D. Participants always observed the outcome of both the chosen and the unchosen option. Importantly, reward probabilities were selected such that the absolute option values were higher for both A compared to C and for B compared to D (Fig. 1); however, the relative values for both A and C, and B and D were identical (0.2 and −0.2, respectively). Thus, an agent learning absolute values ought to clearly prefer option A over C, and option B over D immediately on the first trial of the transfer phase. By contrast, an agent learning relative values would be indifferent between the two options. We refer to these transitions from AB and CD to AC and BD comparisons as TYPE I transitions. Following this transfer phase, participants underwent another acquisition phase with new pairs of stimuli EF and GH. Now, both E and G had an identically high absolute value (for example, reward probability *P*=0.8), but they were either paired with a very poor option (F, *P*=0.2) or a moderately good option (H, *P*=0.6), thus inducing a higher relative value for E compared to G. Participants then entered another transfer phase where they selected between options E and G. This acquisition and transfer procedure was repeated once for a new pair of stimuli. We refer to these transitions as TYPE II transitions. In these TYPE II transfer trials, the opposite behaviour would be expected: an absolute value learner would be indifferent between E and G because both have identical reinforcement histories. A relative value learner however would be biased to prefer E over G, because E has acquired a higher relative value during acquisition (Fig. 1).

### Choices in new context are consistent with relative learning

We tested two cohorts of healthy participants on this task. Experiment 1 was a behavioural experiment, in Experiment 2, participants performed the task during scanning with fMRI. To obtain formal predictions of behaviour on the first transfer trial and to account for individual differences in acquisition learning, we fit two different reinforcement learning models to participants’ behaviour (for modelling details see Supplementary Methods). The first was a Q-learning algorithm that learnt the objective reward probabilities using a simple delta rule (Absolute value learner):




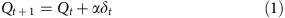




where *Q*_*t*_ is the estimated value on trial *t*, *α* is the participant specific learning rate and *δ*_*t*_=*δ*_C*,t*_ and *δ*_U*,t*_ is the prediction error on trial *t* for the chosen and unchosen option, respectively:




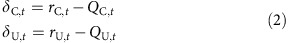




where *r*_C,*t*_ and *r*_U,*t*_ are the rewards (0 or 1) observed on trial *t* on the chosen and unchosen option. The second model (Relative value learner) learnt the relative values of options using the same update rule as in equation (1):




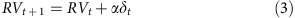




However, here the prediction error *δ*_*t*_ takes the following form:




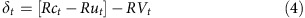




where *Rc*_*t*_ and *Ru*_*t*_ are the outcomes observed on the chosen and unchosen options, respectively. Thus, the outcome difference is compared to the expected outcome difference to update the relative value of options. Note that the Q-learner updates separate option values; on each trial both options of a given pair are updated simultaneously. In contrast, for the relative value learner, there is only one update per trial (for the whole stimulus pair).

Both models make identical predictions about behaviour in the acquisition phase because in both cases, choices are governed by value differences (in the former case, value differences operate at the level of action selection, whereas in the latter case they are part of learning, see Supplementary Methods for details). However, the two models make exactly opposite predictions about choices on the first transfer trial. On TYPE I transitions, an absolute value learner would prefer A over C and B over D because they have higher reward probabilities. In contrast, a relative value learner would be indifferent on these trials, because the options making up the novel combinations AC and BD have identical relative values (Δ*p*=0.2 for both A and C, and Δ*p*=−0.2 for both B and D). In both experiments, participants’ probability to prefer the objectively better option (A or C) on the first trial of TYPE I transitions was not significantly different from chance (*P*=1, Wilcoxon signed rank test, Fig. 1). One might argue that, rather than being a systematic signature of a relative value learning mechanism, this simply reflects an increase in erratic behaviour following the sudden, uninstructed switch to a novel context. Alternatively, if participants had deployed policy learning, they would likewise be required to re-learn a new choice policy when confronted with new combinations. Importantly, our TYPE II transitions control for these issues. On these transitions, the opposite behaviour would be predicted: here, an absolute learner would be indifferent between options E and G, because they had experienced identical reinforcement histories. In contrast, a relative learner would show a preference for option E over G because it had previously been experienced together with a very low value option and thus has a high relative value. In both experiments, we found that participants exhibited a systematic preference for option E on TYPE II transfer trials (Fig. 1, *P*=0.0001 and *P*=0.035 compared to chance, Wilcoxon signed rank test, for Experiments 1 and 2, respectively). Thus, despite the two options having been paired with reward equally often, participants preferred the option that had previously been paired with a lower-value option. The learning models generate choice probabilities for choosing each option on the first trial of each transfer (Fig. 1). From these, we derived model fits (log likelihood estimates, LLE) and Bayes Information Criterion, which we used for comparisons between different models. In both data sets, the relative value learning model provided a significantly better account of behaviour on the first transfer trial than the absolute value learner (mean negative LLE: 1.92 versus 3.34 and 1.88 versus 3.48 for the relative versus absolute learner, in Experiments 1 and 2, respectively, comparison of Bayes Information Criterion: *P*=0.00002 and *P*=0.00018, Wilcoxon signed rank test). The model fits show that, in stark contrast to the relative value learner, the absolute value learner performed below chance levels at predicting choices on the first transfer trial (average model choice probability for selecting the option chosen by the participant=0.62 versus 0.43 and 0.63 versus 0.42 for the relative versus absolute learner in Experiments 1 and 2, respectively). Note that we focused on the decisions on the very first transfer trial, where choices are governed by the previously acquired values (Supplementary Table 1). On subsequent trials, choices are governed by new learning, and absolute and relative value learning will again converge to similar behavioural predictions. Despite this, the relative value learner still tended to perform better compared to the absolute learner when evaluating model fits for the entire transfer period (*P*=0.086 and *P*=0.077 in Experiments 1 and 2, Wilcoxon signed rank test, Supplementary Table 1).

**Figure 2:**
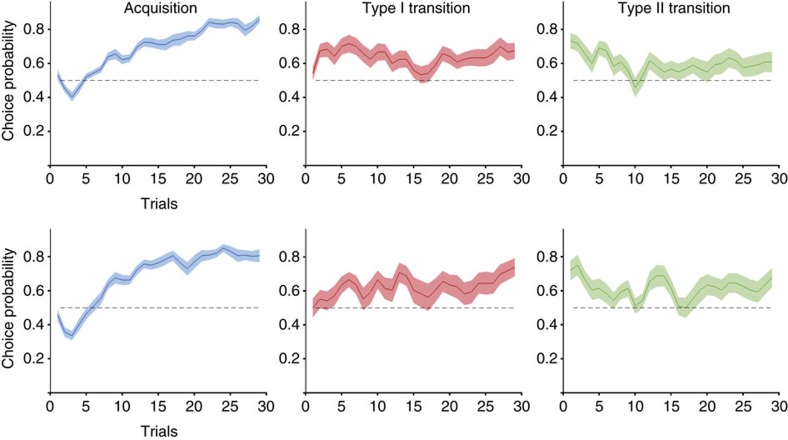
Learning curves for acquisition and transition to new contexts. In both Experiment 1 (top row) and Experiment 2 (bottom row), subjects acquire a robust preference for the objectively higher-value option (left). Following TYPE I transitions to a new context, they are initially indifferent between options, but then gradually re-learn to select the better option, despite occasionally returning to chance levels (middle). Following TYPE II transitions, subjects display a preference for the option with higher relative value, despite identical reinforcement histories (identical absolute value). It takes up to ten trials before this declines to indifference due to new learning. Solid lines represent mean, shaded areas s.e.m. across participants. *x*- and *y* axes are the same across all panels.

The biases upon transfer to a new context had lasting impact (Fig. 2). Following TYPE I transitions, participants took several trials to learn to select the higher-value option, and even though this new learning happened within a few trials, it was somewhat unstable. Following TYPE II transitions, even late in the transfer period, there was still evidence for a subtle preference for the option that previously had acquired a higher relative value. When averaging over the second half of the transfer phase, we find that participants still show a preference for the higher relative value option in Experiment 2, and by trend in Experiment 1 (*P*=0.025 and *P*=0.065, respectively, Wilcoxon signed rank test).

**Figure 3:**
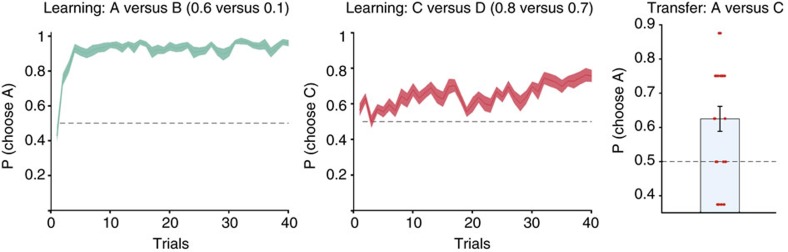
Induction of a systematic preference for the worse option. Using the same experimental setup as in Fig. 1a, subjects first learnt to select the better option in two pairs, AB and CD. Option A had a moderate value (reward probability *P*=0.6), but by virtue of pairing with low-value option B (*P*=0.1) it acquired a high relative value. By contrast, option C had a high absolute value (*P*=0.8), but by virtue of pairing with high-value option D (*P*=0.7), it only acquired a low relative value. Subjects acquired a robust preference for A over B (left) and for C over D (middle). However, when they were suddenly confronted with novel choices between options A and C (right), they systematically preferred the lower-value option A, consistent with relative value learning. Solid lines represent mean, shaded areas s.e.m. across participants.

It is notable that upon transfer to a new context, participants exhibited a systematic preference for one option over an alternative option with identical reinforcement history when the preferred option had previously been paired with a low-value option. This raises the intriguing possibility that, under certain circumstances, participants would systematically prefer an option with a low reward probability over one with a higher value. This should happen when the low value option has been experienced in comparison with a very poor option, and in contrast, the high value option has been experienced in comparison with another high-value option (as in our introductory wine example). In such situations, an option with a lower absolute reward probability would acquire higher relative value and thus should be preferred. We tested this prediction in a third experiment in a new cohort of participants. Here, participants were first exposed to AB and CD discrimination learning. The probabilities were 0.6 versus 0.1 (A versus B) and 0.8 versus 0.7 (C versus D). Participants acquired a robust preference for the better option in both AB and CD pairs (Fig. 3, Supplementary Fig. 2). After this, they were suddenly confronted with a new pairing between A and C. We repeated this entire procedure for eight blocks, using novel stimuli in each block. We found that, when transferred to choices between A and C, participants exhibited a clear preference for the lower value option A over option C on the first transfer trial (*P*=0.0035, Wilcoxon signed rank test, Fig. 3). This propensity to select the objectively worse option, which appears counterintuitive under the assumption of absolute value learning, is exactly the behaviour predicted from an agent learning relative values.

### Alternative learning mechanisms

The behavioural data so far are in favour of our suggestion that participants may learn relative values. However, there are other learning mechanisms that perform a direct comparison between options. In policy gradient methods, no value function is learnt at all. Instead, they operate in policy space^13^,^14^ by directly updating a stochastic choice policy. Such policy learning might be able to explain why performance degrades to chance level upon TYPE I transitions, because a novel comparison is introduced and a new policy has to be learnt. This however cannot explain why participants displayed a preference for the relatively better option upon TYPE II transitions. Thus, our data appear incompatible with policy learning. However, other alternative learning mechanisms are possible (see Supplementary Methods for modelling details). First, one could argue that participants might not have attended properly to the outcomes of the unchosen option. Logistic regression results show that participants’ choices were guided by past outcomes of both the chosen and unchosen option. However, at least in Experiment 1, there was evidence that participants weighted unchosen outcomes somewhat less than chosen outcomes (Supplementary Fig. 1). We therefore set up two simple Q-Learners, one that updates only the value of the chosen option and one that updates both options, but with separate learning rates for the chosen and unchosen option. Both of these Q-Learners clearly failed to reproduce participants’ choices on the first transfer trial (Supplementary Table 1). We also compared our algorithm to a different variant of relative learning^12^. This algorithm differs slightly from the one we present here in that, unlike our algorithm, it does track separate option values. These however are not updated in absolute terms, but relative to an average state value, that in turn is separately updated. Although this state-dependent relative value learner (SD-RVL) was superior compared to our simple RVL during acquisition in Experiment 1 (and by trend in Experiment 2, *P*=0.002 and *P*=0.09, respectively, Wilcoxon signed rank test), it did not provide a good account of choices on the first transfer trial in the new context, the key measure of interest in this study (Supplementary Table 1). This is likely due to the higher learning rate for the chosen versus unchosen option (Supplementary Table 2), which induces a bias towards the chosen option. SD-RVL is conceptually similar to actor-critic learning. The actor-critic architecture we implemented likewise did not provide a good account of participants’ choices in the transfer phase (Supplementary Table 1). Furthermore, it is possible that options are preferred at transfer simply because they had been chosen more frequently during previous acquisition^15^. We tested this using logistic regression to assess whether the relative frequency of choosing one option (for example, A from AB minus C from CD) was predictive of preference (for example, A from AC) in the first transfer trial. We ran this separately for the first transfer trial in each of the four novel combinations. In Experiment 1, there was no effect of choice frequency on bias (*t*-test on the regression weights, *P*>0.25). In Experiment 2, there was only a trend for B versus D choices in predicting preference for B (*t*-test, *P*=0.09). Notably, in the two TYPE II transfer trials where the bias is actually observed, the effect of choice frequency was far from significant (*t*-test, *P*>0.26). Lastly, we wanted to assure participants had learnt all discriminations that were later recombined equally well (for example, whether they were as good at selecting option A from the AC pair as they were at preferring C from CD, prior to exposition to the new combinations AC and BD). We tested the rate at which options had been chosen during the final five trials of acquisition. In Experiment 1, ANOVA revealed no effect of option pair (F_5,145_=1.47, *P*=0.2). In Experiment 2, ANOVA revealed an effect of option pair (F_5,115_=2.8, *P*=0.02). Importantly however, *post-hoc t*-tests showed that this effect was not driven by differences between the option pairs that formed the new combinations during transfer (for example, between AB versus CD which make up the new transfer pairs AC and BD). Furthermore, logistic regression on the average rate of option choices during the last five trials showed no effect of learning success on later bias on the first transfer trial (*P*=0.25 and 0.15 for Experiments 1 and 2, respectively, *t*-test). Furthermore, we ran a separate logistic regression to rule out that any bias on the first transfer trial was due to motor perseveration with regard to the preceding trial. We found no effect of last motor response on choice bias during transfer in any of the three experiments (all *P*>0.25, *t*-test).

### Neural signatures of a relative value prediction error

**Figure 4:**
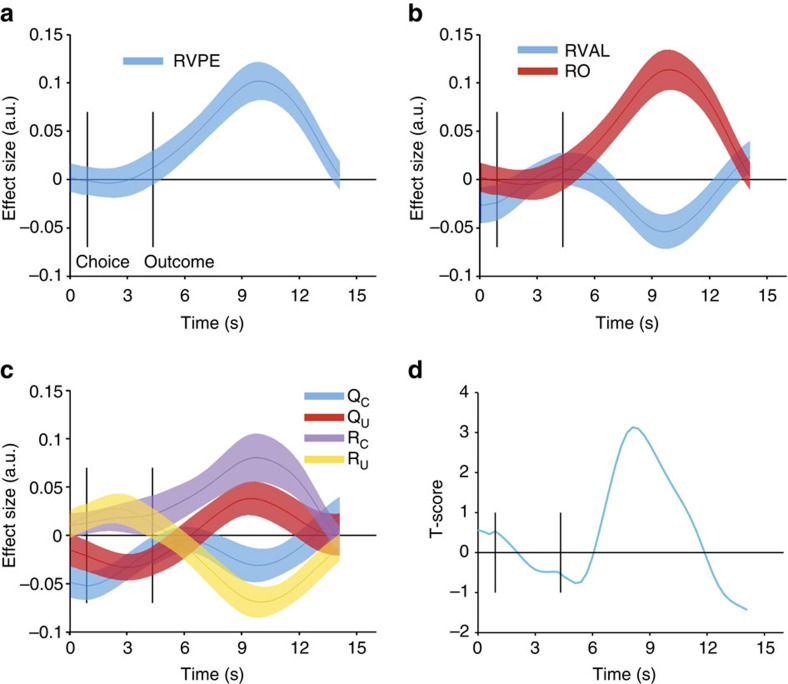
Relative value prediction errors in the striatum. (**a**) Following outcome presentation, BOLD signal (interpolated to a resolution of 300 ms) extracted from a striatal region of interest (see methods for details) correlated with the relative value prediction error. (**b**) The striatal BOLD signal was sensitive to both components of the relative value prediction error, showing the required positive effect of relative outcome (RO) and negative effect of relative value (RVAL). (**c**) Further decomposition revealed that the signal was sensitive to all component terms of a relative value prediction error: it correlated positively with the chosen outcome, *R*_C_ and negatively with chosen value, *Q*_C_—and it correlated negatively with unchosen outcome *R*_U_, and positively with unchosen value, *Q*_U_. Solid lines represent mean, shaded areas s.e.m. of regression coefficients across participants. (**d**) Relationship between striatal relative value coding and relative value-induced biases. Striatal regression coefficients for relative value, relative outcome and response (not shown in **b**) were regressed against model fits (negative log likelihoods) of the relative value learner for the first transfer trial. The figure shows the time course of the resulting T-score for relative value. This indicates that subjects that showed pronounced negative coding of relative value after outcome presentation were also strongly guided by relative values on the first transfer trial (low negative log likelihoods).

Our behavioural data thus far speak to our assumption that participants did indeed learn relative option values. We next sought to investigate whether neural signals also aligned with such a relative value learning mechanism. We focused our analyses on the striatum as the key area where reward prediction errors are thought to drive learning^16^. We extracted BOLD signal time courses from an independent striatal region of interest (ROI, Supplementary Fig. 6A, see Methods), cut it into trial epochs and regressed parameters of interest against this epoched time series in a multiple linear regression (see Methods). The striatal BOLD signal showed a pronounced correlation with the relative value prediction error derived from our model (Fig. 4a, peak *t*_23_=5.3, cluster-corrected *P*=0.0002, permutation test). One hallmark of a prediction error signal is that it is sensitive to both the outcome (here: relative outcome) and expectation term. Specifically, a neural signal that is a prediction error should covary positively with the outcome, but negatively with the expectation term. We therefore set up a separate regression that contained both the expected relative value of the chosen option and the relative outcome (chosen−unchosen outcome). Again, we found a positive correlation with relative outcome (peak *t*_23_=5.53, cluster-corrected *P*<0.00001, permutation test), but also a negative effect of the expectation term (peak *t*_23_=–3.02, cluster-corrected *P*=0.01, permutation test, Fig. 4b). Thus, the signal correlated with both component terms, relative value and relative outcome, of a relative value prediction error. However, it is possible that this effect is exclusively driven by the value and outcome of the chosen option. If the signal truly represents a relative value prediction error, it needs to be sensitive to the components of both the chosen and the unchosen option. In another regression we therefore further decomposed relative value and relative outcome into their component terms: the option values *Q*_C_ and *Q*_U_ for the chosen and unchosen option, and the outcomes *R*_C_ and *R*_U_ on the chosen and unchosen option. Note that during the acquisition phase, relative value is equivalent to the difference between *Q*_C_ and *Q*_U_. We find a positive effect of *R*_C_ and a negative effect of *R*_U_ (peak *t*_23_=3.23 and −4.54, cluster-corrected *P*=0.004 and 0.0006, permutation test, Fig. 4c). We also find a positive effect of *Q*_U_ (peak *t*_23_=2.05, cluster-corrected *P*=0.045, permutation test). In addition, there was a negative effect of *Q*_C_ (peak *t*_23_=2.05), which however did not survive cluster-correction (*P*=0.107, permutation test). Thus, the striatal signal displays the full characteristics of a relative value prediction error. If the signal was used to merely update absolute option values, both outcome and the expectation would have the same sign on the chosen and unchosen option (positive for outcome, negative for expectation). The results reported so far are from an ROI covering parts of all three major striatal subregions. We found little evidence for regional differences within the striatum when we repeated our analyses separately for the ventral striatum, caudate nucleus and putamen (see Supplementary Methods and Supplementary Fig. 7). The only notable difference was that coding of relative value, which was present in both left and right caudate and putamen, was not detectable in the ventral striatum. There was no relative value effect in the right ventral striatum, while in the left ventral striatum, there was an effect that however did not survive cluster-correction (*P*=0.11, permutation test).

**Figure 5:**
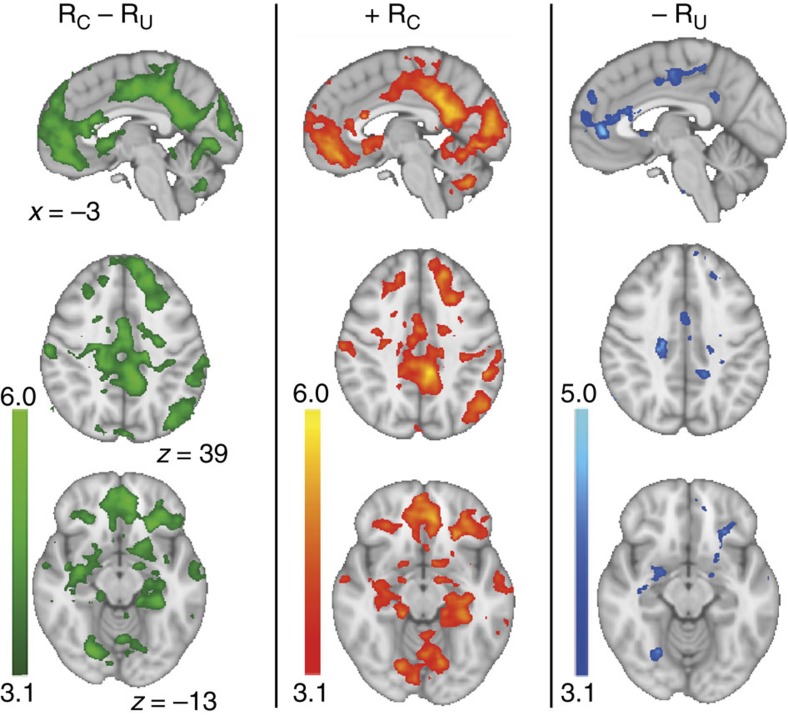
Whole-brain analysis of relative value effects. Many brain regions were sensitive to relative outcome (*R*_C_−*R*_U_, left column). This was driven by both increased responding to rewards on the chosen option (+*R*_C_, middle column) and decreased response to rewards on the unchosen option (–*R*_U_, right column). Colourbars indicate z-scores. Images are thresholded at *z*>3.1.

**Figure 6:**
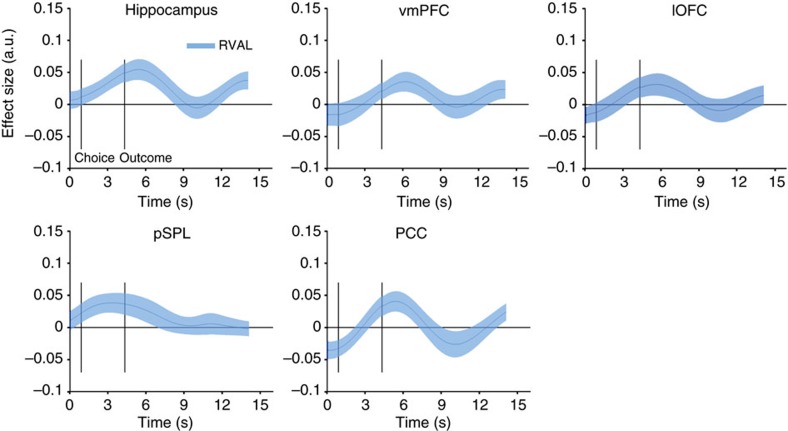
Relative value coding in other brain regions. A whole-brain contrast (see Fig. 5) revealed several areas that positively coded relative outcome. We extracted BOLD signals (interpolated to a resolution of 300 ms) from these areas to test if any of these was also sensitive to relative value (RVAL), thus being candidates for a region coding relative value prediction errors as shown for the striatum in Fig. 4. Solid lines represent mean, shaded areas s.e.m. of regression coefficients across participants. *x*- and *y* axes are the same across all panels.

One could expect that participants in which striatal coding of relative value or relative outcome was very pronounced would also be strongly governed by relative values in their choices on the first transfer trial. We therefore set up a design matrix that contained (along with a constant term) the striatal time courses of regression coefficients for relative value, relative outcome and response (not shown in Fig. 4b) and regressed this matrix against behaviour on the first transfer trial. As dependent variable, we used the model fits of the relative value learner on the first transfer trial, reasoning that participants with very low (negative) log likelihood estimates are the ones who are strongly guided by relative value. We found a positive relation between relative value and the relative learner’s model fit (peak *t*_23_=3.13, cluster-corrected *P*=0.035, permutation test). Specifically, a more pronounced negative effect of relative value following outcome presentation was related to lower log likelihoods (meaning behaviour was strongly guided by relative learning, Fig. 4d). Thus, stronger striatal expression of relative value was related to behaviour being dominated by relative learning. We next asked whether relative value prediction errors were expressed in other brain areas in addition to the striatum. To explore this, a whole-brain contrast of *R*_C_−*R*_U_ (see next section ‘Neural signatures of other learning mechanisms’ and Fig. 5) identified a number of brain regions that responded to relative outcome (increased responding when the chosen option was rewarded and decreased responding to rewards on the non-chosen option). We then tested whether activity in these candidate regions would also correlate negatively with relative value. We thresholded the relative outcome contrast map at *z*>3.1 (we deliberately used a low threshold here to not miss potential regions also showing evidence of relative value learning) and extracted BOLD time series from five ROIs: the ventromedial prefrontal cortex (vmPFC), lateral orbitofrontal cortex (lOFC), hippocampus, posterior cingulate cortex (PCC) and the posterior superior parietal lobule (pSPL). None of these regions showed a significant negative correlation with relative value (cluster-corrected *P*>0.24, Fig. 6) following outcome presentation. Instead, we observed positive coding of relative value following stimulus onset in vmPFC, hippocampus, pSPL and PCC (*P*<0.03, uncorrected for multiple comparisons), and a trend in lOFC (peak *t*_23_=1.82, *P*=0.08, uncorrected).

### Neural signatures of other learning mechanisms

**Figure 7:**
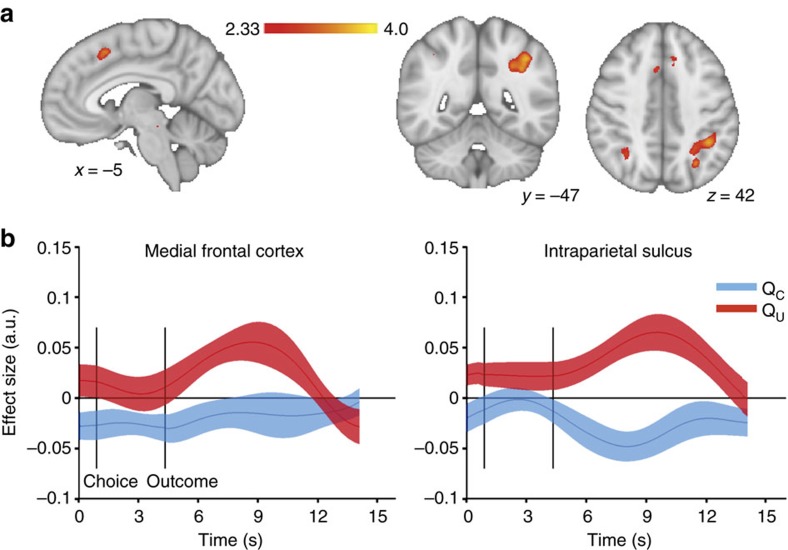
pMFC and IPS outcome signals. (**a**) The opposite pattern as in Fig. 5, an increased response to rewards on the unchosen option was conspicuously absent. Only at a very lenient threshold (*P*<0.01, uncorrected) could we identify regions in pMFC, intraparietal sulcus (IPS) and anterior insula (not shown) that increased responding to rewards on the unchosen option (insula and pMFC additionally responded with signal decrease to chosen outcome, which was not observed for IPS). Therefore, these regions might be candidate regions for learning the value of the non-chosen option. Colourbars indicate *z*-scores. Images are thresholded at Z>2.33. (**b**) However, while the BOLD signal (interpolated to a resolution of 300 ms) in both pMFC and IPS was sensitive to unchosen value, *Q*_U_, following outcome presentation, this effect was pointing in the wrong direction. Both *Q*_U_ and *R*_U_ correlated positively with the BOLD signal in this region, incommensature with a prediction error on the unchosen option. Solid lines represent mean, shaded areas s.e.m. of regression coefficients across participants.

Our results so far show that signals across all three striatal regions display the characteristics of a prediction error that is used to update the relative values of options. We next asked whether there is any region in the brain that might learn option values in absolute terms, that is, an independent estimate of each option’s reward probability. A signal useful for such learning would be required to show the same behaviour on both the chosen and the unchosen option—it needs to correlate positively with the outcome of both the chosen (*R*_C_) and the unchosen option (*R*_U_), but negatively with the expected value of both options (*Q*_C_ and *Q*_U_). Since the effects of expected value are usually less pronounced compared to outcome effects^17^, we first searched for possible candidate regions by looking for regions that showed a positive response to both *R*_C_ and *R*_U_ at the time of outcome presentation. We masked the contrasts for +*R*_C_ and –*R*_U_ at *P*<0.1 and multiplied the resulting maps to obtain a liberal threshold of *P*<0.01, uncorrected. Even at this very lenient threshold, we only found one region in the brain that responded positively to both *R*_C_ and *R*_U_. This region was in the bilateral calcarine sulcus. Further analyses revealed that this region also negatively encoded *Q*_C_ (peak *t*_23_=2.39, cluster-corrected *P*=0.049, permutation test), with no evidence for an effect of *Q*_U_ (peak *t*_23_=1.69, Supplementary Fig. 6). Thus, primary visual cortex was the only region that showed some evidence for an update signal to learn the absolute value of the chosen option, with no evidence for a similar update signal for the unchosen option. Notably, the general pattern that we found was instead that regions that showed increased responding when the chosen option was rewarded deactivated when the unchosen option was rewarded, and vice versa (Fig. 5). Importantly, this cannot simply be explained by an anticorrelation of the two regressors (average correlation across participants following convolution with the haemodynamic response *r*=0.22, see also Supplementary Table 3 and Supplementary Fig. 3 for correlations between all regressors in the design matrix). Instead, it appears that a distributed set of brain regions is responsive to the outcome difference between the chosen and the unchosen option. The opposite pattern, an increased responding to *R*_U_ and decreased responding to *R*_C_ could only be observed in a few regions at a lenient threshold of *P*<0.01 uncorrected. Among these where the insular cortex and a region of the pre-supplementary motor cortex (pre-SMA) in the posterior medial frontal cortex (pMFC, Fig. 7). At this low threshold, an additional area in the region of the intraparietal sulcus (IPS) showed increased responding to *R*_U_, without responding to *R*_C_. Of these regions, the pMFC is of particular interest given the evidence implicating it in representing the value of switching away from the current default option^18^,^19^. We therefore extracted BOLD time series from the peak in the pMFC (MNI *x*=–4, *y*=17, *z*=49) and IPS (MNI *x*=–41, *y*=–45, *z*=44) and performed the same decomposition as in Fig. 4c to test for learning signals in these areas. In both pMFC and IPS there was a positive effect of *Q*_U_ (peak *t*_23_=3.43 and 3.72, cluster-corrected *P*=0.014 and 0.00022, permutation test, Fig. 7b). In addition, there was a negative effect of *Q*_C_ in IPS (peak *t*_23_=3.24, cluster-corrected *P*=0.022, permutation test) but not in pMFC (cluster-corrected *P*=1). Thus, both pMFC and IPS encoded unchosen value and unchosen outcome, but with the same sign (rather than with opposite sign, as would be expected from a prediction error). Hence, pMFC and IPS signals do not fulfil the criteria for being prediction errors to learn either absolute or relative values.

Lastly, we asked whether there were regions in the brain that might encode effector-specific action values. In our task, the side of option presentation was randomized, such that learning action values would not be adaptive. Nevertheless, it is possible that action prediction errors are encoded in the brain, even when not contributing to behaviour. When first running this analysis at whole-brain level, even at a very lenient threshold of *P*<0.01, we found no significant correlation with left or right action value prediction error. We also ran this analysis using an ROI-based approach to directly study two key areas for encoding motor parameters, the primary motor cortex and the sensorimotor striatum. There was no evidence for a prediction error on the left or right option (all *P*>0.31 uncorrected, Supplementary Fig. 8) in the period between 3 and 8 s after the outcome.

## Discussion

We have shown that, upon transfer to a new context, participants’ choices are consistent with the behaviour of an agent who has learnt option values with reference to the previously available alternative options. By creating novel choice contexts, in which objective reward probabilities diverged from the options’ relative values acquired during the previous context, we showed that participants’ choices on the first trials appeared to be governed by relative values. Neural activity in the striatum correlated with a prediction error that is used to update these relative values. While activity in a number of brain regions represented relative reward (the reward difference between the chosen and the unchosen option), the striatum was unique in that it was the only region in which the signal fulfilled the formal criteria of a relative value prediction error. Notably, we found no signal anywhere in the brain that met even the most basic criteria for an update signal that teaches the objective value either of both the chosen and unchosen option, or the unchosen option alone.

In most learning situations, relative and absolute value learning produce identical behaviours. This becomes fundamentally different when previously learnt options are suddenly encountered in a different context. Previous studies have used such transfers to new contexts^9^,^20^,^21^, but usually in a way that relative values were identical to absolute value differences. Here, we created contexts in which absolute and relative value learning made opposite predictions about behaviour on the first trial in the new context (before any new learning could occur). First, participants made choices between two options that had different absolute values (for example, *p*(reward)=0.7 and 0.5), but had previously acquired an identical relative value, because both options had previously been paired with an option that was worse by the same margin. Participants were indifferent on the first trial of the new context, selecting the objectively better option only at chance level, as predicted by a relative learning algorithm. One may argue that the unexpected transfer to a new context caused participants’ choices to become erratic. Alternatively, policy gradient methods^13^ might also explain chance-level performance on the first transfer trial, since it appears natural that a new context requires establishing a new policy. However, with our TYPE II transfers, we had another condition in which participants showed, on the first trial in the new context, a systematic preference for one option over the other, despite the two previously having received identical reinforcement histories (that is, same absolute values). The option preferred by participants, however, had previously acquired a higher relative value by virtue of pairing with a lower-value alternative option. This appears hard to explain by erratic behaviour or by re-setting of the choice policy upon transfer to a new context. Even more strikingly, in a further experiment we show that participants systematically preferred an option that had a lower absolute value compared to the alternative option when this lower value option had previously acquired a higher relative value. We tried to rule out a number of alternative explanations, such as diminished attention to the outcomes of the unchosen option. We show that participants’ choices were guided by outcomes of both the chosen and unchosen options. Furthermore, absolute learning models updating either only the chosen option, or both options but with separate learning rates, were not able to reproduce the behavioural pattern upon transfer to a new context. The same was true for actor-critic learning.

Deviations from economically rational behaviour are well-known in the literature on economic and perceptual decision making^22^,^23^,^24^,^25^. While a number of studies have investigated the neural underpinnings of these so-called cognitive biases, these studies focus on the decision-making process using options with values known to participants. However, decision makers often have to estimate option values from experience. Reinforcement learning models offer a powerful framework for studying this learning from experience. It has recently been shown that relative value learning can account for efficient avoidance learning (providing a solution to a computational paradox)^12^. Importantly, this study also suggests that seemingly irrational economic decisions can arise as a consequence of context-dependent relative value learning. Behavioural ecology has described examples of context-, or state-dependent learning in a number of species^26^,^27^,^28^. Honeybees were more likely to leave a reward source that contained a 20% sucrose solution when they had previously been trained to expect a 50% sucrose solution, compared to control animals that had only experienced the 20% solution^29^. It has been suggested that behaviour that appears suboptimal in simplified laboratory settings arises as a consequence of decision rules that have been favoured by natural selection because they are beneficial in naturalistic environments^30^,^31^,^32^. Here, we apply algorithmic models of reinforcement learning to account for such context-dependent learning in humans. We show that when information about both options is available, humans appear to learn option values relative to the available alternatives. Our data do not necessarily generalize to situations where outcome information is provided only for the chosen option. A recent study comparing such partial feedback contexts with complete feedback showed that while relative value learning was more pronounced in complete feedback contexts, it was also present in the partial feedback context, hinting at the possibility that relative value encoding might generalize to partial feedback contexts^12^. Another study using a similar design but only partial feedback likewise found behaviour that was not compatible with learning of objective values^33^.

We have used a very simple approach to model relative value learning by substituting the outcome difference for absolute outcome in standard Q-learning algorithms. There are other ways of implementing a relative learner. Palminteri and colleagues^12^ used an algorithm that learnt the average value of the current context (state value) and then (unlike in our approach) separate option values were learnt with reference to that state value. While this approach was less successful than our algorithm at predicting choices in the new context, we explicitly refrain from making claims about what would be the best relative value learning model. Our neural data are incompatible with learning of separate option values, even when they are referenced to state value, since both the expectation and the outcome term of the unchosen option were encoded with reversed sign compared to the chosen option, which is indicative of a signal that encodes the direct relative advantage of one option over the other. This is consistent with Palminteri and colleagues showing that the striatal signal correlates positively with the chosen prediction error, but negatively with the unchosen prediction error^12^. The exact algorithm used by participants is however likely to vary with specifics of the task at hand.

Activity in the striatum at the time when the outcomes of the two options were revealed exhibited the characteristics of a relative value prediction error. The BOLD signal encoded all component terms of a relative value prediction error with the appropriate sign. Whereas a recent study reporting a risk-sensitive prediction error found the ventral striatum to be unique in responding to all components of a prediction error^34^, we found no evidence for a differential pattern of responses between the three major striatal subregions. The properties of the striatal signal were somewhat unique: while several regions in the brain correlated positively with relative outcome, none of these regions exhibited the characteristics of a relative value prediction error. Notably, even at a very liberal threshold, we found brain region that showed characteristics of a prediction error for learning absolute values of both options independently. Similarly, we could not find any signals related to updating the absolute value of the unchosen option alone. Few areas in the brain like pMFC, insula and IPS showed increased responding to rewards on the unchosen option (at a very lenient threshold). However, none of these areas showed a negative effect of unchosen value. Taken together, we find a number of brain regions that exhibited relative outcome effects, with the striatum encoding a relative value prediction error. By contrast, we unexpectedly found no signal anywhere in the brain that might be useful for updating absolute option values. One explanation may be that absolute values and outcomes are only transiently represented in the brain and rapidly transformed into relative values and relative outcomes. Such transient network states would be invisible with fMRI, but might be detected using the high temporal resolution of MEG^35^. Thus, it still appears possible that both absolute and relative values are updated simultaneously, consistent with the notion of multiple systems for learning and memory operating in parallel^36^,^37^,^38^,^39^,^40^,^41^. One recent study found that the striatal signal correlated with relative outcome, corresponding to a policy prediction error, with no representation of either chosen or unchosen value anywhere in the brain, not even at liberal thresholds^14^. This is surprising since the task they used is essentially identical to the acquisition phase of our task. One reason for this discrepancy may lie in the analysis approaches used (for example, whole-brain versus ROI).

Our results also bear some resemblance to a fictive error signal described in the striatum^42^ which represented the difference between reward obtained and the maximum possible reward. This fictive error however corresponds to a regret signal. This is not the case in our study, where the striatal signal not only correlates negatively with foregone rewards but also positively with the expected value of the unchosen option. One question arising from the relative value coding scheme we found pertains to what the frame of reference would be in multi-alternative choice contexts, where there is more than one unchosen option. One study suggests that in such situations, learning might occur relative to an estimated next-best option^43^.

Taken together, both our behavioural data and the striatal BOLD signal align with the predictions of an algorithm learning the value of options relative to the available alternatives. Relative value signals have been well described in the decision-making literature, where it is often found that vmPFC activity correlates with the difference between the chosen and unchosen option value^44^,^45^,^46^,^47^,^48^. Similarly, a number of studies suggests that value representations are normalized by the range of available alternatives (for review see ref. 49), similar to divisive normalization in sensory systems^50^. Decision making is often influenced by the context in which decisions are made. Our results take this one step further to suggest that seemingly irrational decisions may arise because even the learning of option values is already dependent on the context in which these options are encountered during learning.

## Methods

### Participants

We tested *n*=30 participants (age: mean±s.d. 25.07±3.1 years, *n*=15 female) in experiment 1, *n*=24 participants (age: 26.21±3.7, *n*=12 female) in experiment 2, and *n*=21 participants (age: 25.04±2.4, *n*=12 female) in experiment 3. All participants were healthy volunteers and gave written informed consent before participation. The procedures were conducted in accordance with the Declaration of Helsinki and the guidelines of the ethics committee of the University of Leipzig.

### Behavioural task

We used a reinforcement learning task in which participants repeatedly made choices between two abstract symbols that were probabilistically associated with reward. Participants’ task was to learn, by trial and error, to maximize their rewards by selecting the option with the higher reward probability. Each trial started with presentation of the two options until the participant responded by pressing a button with the index finger of the left or right hand, respectively. If no response was registered within 1.5 s, a prompt appeared instructing participants to respond faster. In the case of such missed responses, the trial was repeated to ensure that participants were able to learn the underlying reward probabilities prior to being switched to a new context. Once a response was made, the choice was confirmed by a thickening of the grey frame around the chosen option. This remained onscreen until presentation of the outcome, which always occurred 3.5 s after the choice. The outcome was indicated by a change of the colour of the frame around the option. When an option was rewarded, the frame turned green, when it was not rewarded, it turned red. Participants were instructed that they could earn a bonus of up to €8 (each reward obtained on the chosen option yielded €0.03), depending on how often their choices resulted in reward. Furthermore, they were also instructed that the outcome of the unchosen option had no financial consequences for them, but that they could nevertheless use these outcomes to learn about ‘how good’ the other option was. Logistic regression (Supplementary Fig. 1) showed that participants used the outcomes of both the chosen and unchosen option to guide their choices. The outcome was displayed for 1.5 s, which was followed by an intertrial interval (blank grey screen, 2–4 s) before the next trial began. We ran the task twice, once as a behavioural study (Experiment 1) and once while scanning with fMRI (Experiment 2). In Experiment 1, trial timing was faster than in the fMRI study: the delay between choice and outcome was set to 1 s, the intertrial interval was 1 s. In a third experiment, we ran a modified version of the task (see below) as behavioural study.

Importantly, the task consisted of acquisition and transfer stages. In a first acquisition stage, participants made choices between symbols pairs AB and CD that were presented in random order for 30 trials each. The reward probability for A and B was *P*=0.7 and *P*=0.5, the probability for C and D was *P*=0.5 and *P*=0.3. After this acquisition, subjects entered the transfer stage, where they were presented with novel combinations AC and BD for 30 trials. Participants were not informed of this change of context. Participants were presented with trial outcomes during the transfer stage just as they were during the first stage, allowing them to continually learn option values. An agent learning absolute option values would exhibit a preference for A over C (*P*=0.7 versus 0.5) and for B over D (*P*=0.5 versus 0.3) on the first transfer trial. By contrast, an agent learning relative values would be indifferent on the first transfer trial, because in both AC and BD trials, both options had acquired the same relative value (RV=0.2 for A and C, RV=−0.2 for B and D). We refer to these transfers as TYPE I transitions (differing absolute value, same relative value). This was followed by another acquisition phase where subjects were presented with pairs EF and GH. Here, the probabilities were *P*=0.7 and 0.2 (EF) and *P*=0.7 and 0.5 (GH). After 30 trials, subjects entered another transfer stage where they now made choices between the novel combination EG. We repeated this procedure once with new stimuli for EF and GH, where the probabilities were 0.8 versus 0.3 (EF) and 0.8 versus 0.6 (GH). We refer to the two sets of EF and GH stimuli as EF(1) and GH(1), and EF(2) and GH(2), respectively, in Supplementary Figs 4 and 5. Here, an agent learning absolute values ought to be indifferent between E and G on the first transfer trial, because both options have identical reinforcement histories (*P*=0.7 or *P*=0.8, respectively). In contrast, an agent learning relative values should start the transfer stage with a preference for E, because it acquired a higher relative value compared to G during the learning phase (for example, RV=0.5 for E versus RV=0.2 for G). We refer to this kind of transfer as TYPE II transitions (same absolute value, differing relative value). While in TYPE I transitions, both stimuli from each pair could be used to create a novel combination of options of the same relative value (AC and BD), from the pairs EF and GH we could only use E and G to create one novel pair of options with different relative (but same absolute) value. Thus, in order to have two TYPE II transitions, we repeated this procedure once with a novel set of stimuli EF and GH (see above). Thus, there were two transitions for both TYPE I (AC and BD trials) and TYPE II (EG1 and EG2 trials) transitions. Experiment 3 had the same structure of acquisition and transfer stages. However, here probabilities were structured such that upon transfer, relative values would mandate choice of the option with a lower absolute value. In particular, subjects learnt to select the better option from pairs AB (*P*=0.6 and 0.1) and CD (*P*=0.8 and 0.7) for 40 trials for each pair. Thereafter, they performed ten transfer trials, where they had to select between options A and C. This procedure was repeated seven times using novel stimuli in each block. An agent learning absolute values would prefer option C over A, because of its higher objective value (*P*=0.8 versus 0.6). An agent learning relative values however would systematically prefer the objectively less valuable option A, since it has acquired a higher relative value than C (relative value=0.5 versus 0.1).

### Acquisition and analysis of MRI data

MRI data were acquired on a 3T Siemens Magnetom Trio system equipped with a 12 channel phased array head coil. Forty-five slices (3 mm thickness, no interslice gap) were obtained tilted with an angle of 30° with respect to the anterior commissure–posterior commissure line using a single-shot gradient echo-planar imaging (EPI) sequence (repetition time, 3,000 ms; echo time, 30 ms; bandwidth, 116 kHz; flip angle, 90°; 64 × 64 pixel matrix; field of view, 192 mm) sensitive to blood oxygen level-dependent (BOLD) contrast. A total of 994 volumes were acquired on average, depending on subjects’ reaction times and number of trials missed, thus resulting in a total task duration of about 50 min. For B0 unwarping of the EPI images, field maps were acquired using a gradient echo sequence (TR: 1,260 ms; TE: 5.20, 9.39 and 15.38 ms; flip angle: 60°; 128 × 128 pixel matrix, FOV: 210 mm) of the same geometry as the EPI images. To improve localization, a high resolution anatomical brain image was recorded from each participant in a separate session using a modified driven equilibrium Fourier transform sequence. We used Presentation (Neurobehavioural Systems, USA) to present the task and record subjects’ behaviour.

Analysis of fMRI data was performed using tools from the Functional Magnetic Resonance Imaging of the Brain (FMRIB) Software Library, FSL^51^. Functional data were motion-corrected using rigid-body registration to the central volume^52^, corrected for geometric distortions using the field maps and an n-dimensional phase-unwrapping algorithm^53^, high-pass filtered using a Gaussian-weighted lines filter (1/100 Hz) and spatially smoothed using a Gaussian filter with 6 mm full-width at half maximum. EPI images were registered with the high-resolution brain images and normalized into standard (MNI) space using affine registration^54^. A general linear model was fitted into prewhitened data space to account for local autocorrelations^55^. For whole-brain analysis, we set up a single GLM that contained two regressors that coded for the outcome (reward or non-reward) on the chosen and unchosen option (modelled at outcome onset) and four regressors coding for the main effects of stimulus presentation (modelled at stimulus onset), outcome presentation (modelled at outcome onset) and left and right responses (modelled as stick functions at response onset). In addition, the six motion parameters from the motion correction were included in the model to account for residual head motion. Contrasts were computed for the chosen and unchosen outcomes, and for the difference between chosen and unchosen outcomes. This served to identify candidate regions for other kinds of learning signals (shown in Fig. 7). Additionally, we contrasted right versus left responses to identify brain regions coding motor parameters. Contrast images from the first level were then taken to the group level using a random effects analysis. Results are reported at *P*<0.001 uncorrected, unless stated otherwise. Note that we deliberately applied a lenient threshold. This is because these analyses were designed to identify regions that did not conform with our hypothesis of relative value learning and instead might exhibit absolute value learning.

### ROI analyses

To obtain an independent functional ROI of the striatum, we used the *z*-map of prediction error effects from a previous study^9^ (data taken from placebo condition), thresholded it at *Z*>2.3 and multiplied the resulting image with anatomical masks from the Harvard–Oxford subcortical atlas for the caudate nucleus, putamen and ventral striatum of the left and right hemisphere. We merged these masks together to yield one mask for the entire striatum, in addition to the six subregion- and hemisphere-specific masks (Supplementary Fig. 6A). The putaminal parts of this mask were additionally used for the analysis searching for action value prediction errors (Supplementary Fig. 8). A ROI for left and right motor cortex was obtained by thresholding the contrast image for right versus left responses at *z*>5.0. In addition, we extracted data from the peak of MFC and IPS that responded to unchosen outcomes, as described in the results section. The BOLD data from these regions were then processed as in previous studies^10^,^47^,^48^ according to the following procedures: Using custom-written Matlab routines, the time series of each volunteer was first cut into trials with a duration of 14.4 s, where options were presented at 0 s, the response was made at 0.9 s (the mean response time across subjects) and the outcome was presented at 4.32 s, which corresponds to the mean onsets of these events across subjects and trials. Time series were resampled to a resolution of 300 ms using cubic spline interpolation. Thus, the resulting data matrix was of size *m* × *n*, where *m*=number of trials and *n*=number of time points. A GLM containing the parameters of interest was then fitted at each time point for each participant (across trials). Note that regressors are not modelled at any specific onset; instead the entire GLM is fitted at each time point in a trial to the data of all trials at this time point. This resulted in a time course of effect sizes for each regressor in the design matrix and for each participant. These time courses were then averaged across participants. A first GLM was set up that contained (along with a constant) only the relative value prediction error. Further GLMs were then set up to further examine the relative value prediction error in greater detail. First, the prediction error was decomposed into its constituent terms, relative value and relative outcome, using separate regressors. Finally, both of these terms were further decomposed into *Q*_C_ and *Q*_U_ (relative value) and *R*_C_ and *R*_U_ (relative outcome). A separate analysis was designed to search for potential action value signal. This GLM included (along with a constant) two regressors coding the action values for the left and right option, and two regressors coding for the outcome on the left and right option (for the sake of clarity, we show the effects of action value prediction error for the left and right option, without further splitting up the prediction error into left and right action values and outcomes). In all models, the response (left or right) made by the subject and the (log) response time on each trial was included as additional covariate. The time courses of regression coefficients were tested for significant effects using cluster-based random permutation testing. The time series of regression coefficients was randomly sign-flipped, a t-statistic was computed, and t-scores were summed in contiguous clusters along the time dimension significant at *α*=0.05. This was repeated 5,000 times and the largest cluster in each iteration was kept to generate a null distribution of significant clusters. This was then compared to the largest cluster observed empirically in our data. As in previous work^10^, we considered the time between 4 and 8 s post-outcome for statistical testing, corresponding to the window during which the BOLD response is expected to peak.

### Data availability

The authors declare that all the behavioural raw data that were used in this manuscript are included in the Supplementary Information. The fMRI data sets generated in this study and code used to analyse the data are available from the corresponding author on reasonable request.

## Additional information

**How to cite this article:** Klein, T. A. *et al*. Learning relative values in the striatum induces violations of normative decision making. *Nat. Commun.* **8**, 16033 doi: 10.1038/ncomms16033 (2017).

**Publisher’s note:** Springer Nature remains neutral with regard to jurisdictional claims in published maps and institutional affiliations.

## Supplementary Material

Supplementary Information

## References

[b1] Sutton R. S. & Barto A. G. Reinforcement learning: an introduction MIT Press (1998).

[b2] Schultz W., Dayan P. & Montague P. R. A neural substrate of prediction and reward. Science 275, 1593–1599 (1997). 9054347 10.1126/science.275.5306.1593

[b3] Shen W., Flajolet M., Greengard P. & Surmeier D. J. Dichotomous dopaminergic control of striatal synaptic plasticity. Science 321, 848–851 (2008). 18687967 10.1126/science.1160575 PMC2833421

[b4] Steinberg E. E. . A causal link between prediction errors, dopamine neurons and learning. Nat. Neurosci. 16, 966–973 (2013). 23708143 10.1038/nn.3413 PMC3705924

[b5] O’Doherty J. . Dissociable roles of ventral and dorsal striatum in instrumental conditioning. Science 304, 452–454 (2004). 15087550 10.1126/science.1094285

[b6] O’Doherty J. P., Dayan P., Friston K., Critchley H. & Dolan R. J. Temporal difference models and reward-related learning in the human brain. Neuron 38, 329–337 (2003). 12718865 10.1016/s0896-6273(03)00169-7

[b7] Pagnoni G., Zink C. F., Montague P. R. & Berns G. S. Activity in human ventral striatum locked to errors of reward prediction. Nat. Neurosci. 5, 97–98 (2002). 11802175 10.1038/nn802

[b8] Pessiglione M., Seymour B., Flandin G., Dolan R. J. & Frith C. D. Dopamine-dependent prediction errors underpin reward-seeking behaviour in humans. Nature 442, 1042–1045 (2006). 16929307 10.1038/nature05051 PMC2636869

[b9] Jocham G., Klein T. A. & Ullsperger M. Dopamine-mediated reinforcement learning signals in the striatum and ventromedial prefrontal cortex underlie value-based choices. J. Neurosci. 31, 1606–1613 (2011). 21289169 10.1523/JNEUROSCI.3904-10.2011 PMC6623749

[b10] Jocham G., Klein T. A. & Ullsperger M. Differential modulation of reinforcement learning by D2 dopamine and NMDA glutamate receptor antagonism. J. Neurosci. 34, 13151–13162 (2014). 25253860 10.1523/JNEUROSCI.0757-14.2014 PMC4262707

[b11] Breiter H. C., Aharon I., Kahneman D., Dale A. & Shizgal P. Functional imaging of neural responses to expectancy and experience of monetary gains and losses. Neuron 30, 619–639 (2001). 11395019 10.1016/s0896-6273(01)00303-8

[b12] Palminteri S., Khamassi M., Joffily M. & Coricelli G. Contextual modulation of value signals in reward and punishment learning. Nat. Commun. 6, 8096 (2015). 26302782 10.1038/ncomms9096 PMC4560823

[b13] Dayan P. & Daw N. D. Decision theory, reinforcement learning, and the brain. Cogn. Affect Behav. Neurosci. 8, 429–453 (2008). 19033240 10.3758/CABN.8.4.429

[b14] Li J. & Daw N. D. Signals in human striatum are appropriate for policy update rather than value prediction. J. Neurosci. 31, 5504–5511 (2011). 21471387 10.1523/JNEUROSCI.6316-10.2011 PMC3132551

[b15] Cockburn J., Collins A. G. & Frank M. J. A reinforcement learning mechanism responsible for the valuation of free choice. Neuron 83, 551–557 (2014). 25066083 10.1016/j.neuron.2014.06.035 PMC4126879

[b16] Stuber G. D. . Reward-predictive cues enhance excitatory synaptic strength onto midbrain dopamine neurons. Science 321, 1690–1692 (2008). 18802002 10.1126/science.1160873 PMC2613864

[b17] Behrens T. E., Hunt L. T., Woolrich M. W. & Rushworth M. F. Associative learning of social value. Nature 456, 245–249 (2008). 19005555 10.1038/nature07538 PMC2605577

[b18] Boorman E. D., Rushworth M. F. & Behrens T. E. Ventromedial prefrontal and anterior cingulate cortex adopt choice and default reference frames during sequential multi-alternative choice. J. Neurosci. 33, 2242–2253 (2013). 23392656 10.1523/JNEUROSCI.3022-12.2013 PMC3743024

[b19] Kolling N., Behrens T. E., Mars R. B. & Rushworth M. F. Neural mechanisms of foraging. Science 336, 95–98 (2012). 22491854 10.1126/science.1216930 PMC3440844

[b20] Klein T. A. . Genetically determined differences in learning from errors. Science 318, 1642–1645 (2007). 18063800 10.1126/science.1145044

[b21] Frank M. J., Seeberger L. C. & O’Reilly R. C. By carrot or by stick: cognitive reinforcement learning in parkinsonism. Science 306, 1940–1943 (2004). 15528409 10.1126/science.1102941

[b22] De Martino B., Kumaran D., Seymour B. & Dolan R. J. Frames, biases, and rational decision-making in the human brain. Science 313, 684–687 (2006). 16888142 10.1126/science.1128356 PMC2631940

[b23] Tsetsos K., Chater N. & Usher M. Salience driven value integration explains decision biases and preference reversal. Proc. Natl Acad. Sci. USA 109, 9659–9664 (2012). 22635271 10.1073/pnas.1119569109 PMC3386128

[b24] Tsetsos K., Usher M. & Chater N. Preference reversal in multiattribute choice. Psychol. rev. 117, 1275–1293 (2010). 21038979 10.1037/a0020580

[b25] Trueblood J. S., Brown S. D., Heathcote A. & Busemeyer J. R. Not just for consumers: context effects are fundamental to decision making. Psychol. sci. 24, 901–908 (2013). 23610134 10.1177/0956797612464241

[b26] Aw J. M., Holbrook R. I., Burt de Perera T. & Kacelnik A. State-dependent valuation learning in fish: banded tetras prefer stimuli associated with greater past deprivation. Behav. process. 81, 333–336 (2009). 10.1016/j.beproc.2008.09.002 18834933

[b27] Pompilio L. & Kacelnik A. State-dependent learning and suboptimal choice: when starlings prefer long over short delays to food. Anim. Behav. 70, 571–578 (2005).

[b28] Pompilio L., Kacelnik A. & Behmer S. T. State-dependent learned valuation drives choice in an invertebrate. Science 311, 1613–1615 (2006). 16543461 10.1126/science.1123924

[b29] Couvillon P. A. & Bitterman M. E. The overlearning-extinction effect and successive negative contrast in honeybees (Apis mellifera). J. comp. psychol. 98, 100–109 (1984). 6705502

[b30] McNamara J. M., Trimmer P. C. & Houston A. I. The ecological rationality of state-dependent valuation. Psychol. rev. 119, 114–119 (2012). 22022832 10.1037/a0025958

[b31] McNamara J. M., Fawcett T. W. & Houston A. I. An adaptive response to uncertainty generates positive and negative contrast effects. Science 340, 1084–1086 (2013). 23723234 10.1126/science.1230599

[b32] Fawcett, Tim W.. . The evolution of decision rules in complex environments. Trends Cogn. Sci. 18, 153–161 (2014). 24467913 10.1016/j.tics.2013.12.012

[b33] Gold J. M. . Negative symptoms and the failure to represent the expected reward value of actions: behavioral and computational modeling evidence. Arch. Gen. Psychiatry 69, 129–138 (2012). 22310503 10.1001/archgenpsychiatry.2011.1269 PMC4406055

[b34] Niv Y., Edlund J. A., Dayan P. & O’Doherty J. P. Neural prediction errors reveal a risk-sensitive reinforcement-learning process in the human brain. J. Neurosci. 32, 551–562 (2012). 22238090 10.1523/JNEUROSCI.5498-10.2012 PMC6621075

[b35] Hunt L. T. . Mechanisms underlying cortical activity during value-guided choice. Nat. Neurosci. 15, 470–476 S471–473 (2012). 22231429 10.1038/nn.3017 PMC3378494

[b36] Belin D., Jonkman S., Dickinson A., Robbins T. W. & Everitt B. J. Parallel and interactive learning processes within the basal ganglia: relevance for the understanding of addiction. Behav. brain res. 199, 89–102 (2009). 18950658 10.1016/j.bbr.2008.09.027

[b37] Daw N. D., Niv Y. & Dayan P. Uncertainty-based competition between prefrontal and dorsolateral striatal systems for behavioral control. Nat. Neurosci. 8, 1704–1711 (2005). 16286932 10.1038/nn1560

[b38] Knowlton B. J., Mangels J. A. & Squire L. R. A neostriatal habit learning system in humans. Science 273, 1399–1402 (1996). 8703077 10.1126/science.273.5280.1399

[b39] McDonald R. J., Devan B. D. & Hong N. S. Multiple memory systems: the power of interactions. Neurobiol. Learn Mem. 82, 333–346 (2004). 15464414 10.1016/j.nlm.2004.05.009

[b40] Poldrack R. A. . Interactive memory systems in the human brain. Nature 414, 546–550 (2001). 11734855 10.1038/35107080

[b41] Jocham G. . Reward-guided learning with and without causal attribution. Neuron 90, 177–190 (2016). 26971947 10.1016/j.neuron.2016.02.018 PMC4826429

[b42] Lohrenz T., McCabe K., Camerer C. F. & Montague P. R. Neural signature of fictive learning signals in a sequential investment task. Proc. Natl Acad. Sci. USA 104, 9493–9498 (2007). 17519340 10.1073/pnas.0608842104 PMC1876162

[b43] Boorman E. D., Behrens T. E. & Rushworth M. F. Counterfactual choice and learning in a neural network centered on human lateral frontopolar cortex. PLoS Biol. 9, e1001093 (2011). 21738446 10.1371/journal.pbio.1001093 PMC3125157

[b44] FitzGerald T. H., Seymour B. & Dolan R. J. The role of human orbitofrontal cortex in value comparison for incommensurable objects. J. Neurosci. 29, 8388–8395 (2009). 19571129 10.1523/JNEUROSCI.0717-09.2009 PMC2712081

[b45] Philiastides M. G., Biele G. & Heekeren H. R. A mechanistic account of value computation in the human brain. Proc. Natl Acad. Sci. USA 107, 9430–9435 (2010). 20439711 10.1073/pnas.1001732107 PMC2889112

[b46] Boorman E. D., Behrens T. E., Woolrich M. W. & Rushworth M. F. How green is the grass on the other side? Frontopolar cortex and the evidence in favor of alternative courses of action. Neuron 62, 733–743 (2009). 19524531 10.1016/j.neuron.2009.05.014

[b47] Jocham G., Hunt L. T., Near J. & Behrens T. E. A mechanism for value-guided choice based on the excitation-inhibition balance in prefrontal cortex. Nat. Neurosci. 15, 960–961 (2012). 22706268 10.1038/nn.3140 PMC4050076

[b48] Jocham G. . Dissociable contributions of ventromedial prefrontal and posterior parietal cortex to value-guided choice. Neuroimage 100, 498–506 (2014). 24941453 10.1016/j.neuroimage.2014.06.005 PMC4148525

[b49] Rangel A. & Clithero J. A. Value normalization in decision making: theory and evidence. Curr. Opin. Neurobiol. 22, 970–981 (2012). 22939568 10.1016/j.conb.2012.07.011 PMC4334383

[b50] Carandini M. & Heeger D. J. Normalization as a canonical neural computation. Nat. Rev. Neurosci. 13, 51–62 (2012). 10.1038/nrn3136 PMC3273486 22108672

[b51] Smith S. M. . Advances in functional and structural MR image analysis and implementation as FSL. Neuroimage 23, (Suppl 1): S208–S219 (2004). 15501092 10.1016/j.neuroimage.2004.07.051

[b52] Jenkinson M., Bannister P., Brady M. & Smith S. Improved optimization for the robust and accurate linear registration and motion correction of brain images. Neuroimage 17, 825–841 (2002). 12377157 10.1016/s1053-8119(02)91132-8

[b53] Jenkinson M. Fast, automated, N-dimensional phase-unwrapping algorithm. Magn. Reson. Med. 49, 193–197 (2003). 12509838 10.1002/mrm.10354

[b54] Jenkinson M. & Smith S. A global optimisation method for robust affine registration of brain images. Med. Image Anal. 5, 143–156 (2001). 11516708 10.1016/s1361-8415(01)00036-6

[b55] Woolrich M. W., Ripley B. D., Brady M. & Smith S. M. Temporal autocorrelation in univariate linear modeling of FMRI data. Neuroimage 14, 1370–1386 (2001). 11707093 10.1006/nimg.2001.0931

