# Publisher Correction: Learning relative values in the striatum induces violations of normative decision making

**DOI:** 10.1038/s41467-019-10718-8

**Published:** 2019-06-27

**Authors:** Tilmann A. Klein, Markus Ullsperger, Gerhard Jocham

**Affiliations:** 1 Cognitive Neuroscience, Center for Behavioral Brain Sciences, Otto-von-Guericke-Universität Magdeburg, Universitätsplatz 2, 39106 Magdeburg, Germany; 2 Institute of Psychology, Otto-von-Guericke-Universität Magdeburg, Universitätsplatz 2, 39106 Magdeburg, Germany; 3 Center for Behavioral Brain Sciences, Otto-von-Guericke-Universität Magdeburg, Universitätsplatz 2, 39106 Magdeburg, Germany; 4 Max Planck Institute for Human Cognitive and Brain Sciences, Stephanstraβe 1a, 04103 Leipzig, Germany; 5 Day Clinic for Cognitive Neurology, University Hospital Leipzig, Liebigstraβe 16, 04103 Leipzig, Germany

Correction to: *Nature Communications* 10.1038/ncomms16033, published online 20 June 2017.

This Article contains an error in the first sentence of the ‘Data availability’ section of the “Methods”, which incorrectly reads ‘The authors declare that all the behavioural raw data that were used in this manuscript are included in the Supplementary Information.’ This should read ‘The authors declare that all the behavioural raw data that were used in this manuscript are included in Supplementary Data 1.’ The error has not been fixed in the PDF or HTML versions of the Article.

Additionally, the HTML version of this Article incorrectly omits Supplementary Data 1 file. Supplementary Data 1 can be found as Supplementary Information associated with this Correction.

## Electronic supplementary material

Supplementary Figures

