# Supplementary material for: Learning relative values in the striatum induces violations of normative decision making
Source: Nat Commun. 2017 Jun 20;8:16033. doi: 10.1038/ncomms16033 (PMC5481835; doi:10.1038/ncomms16033)
Supplement: Supplementary Information [file ncomms16033-s1.pdf]

Title of file for HTML: Supplementary Information
Description: Supplementary Figures, Supplementary Tables, Supplementary Methods and Supplementary References

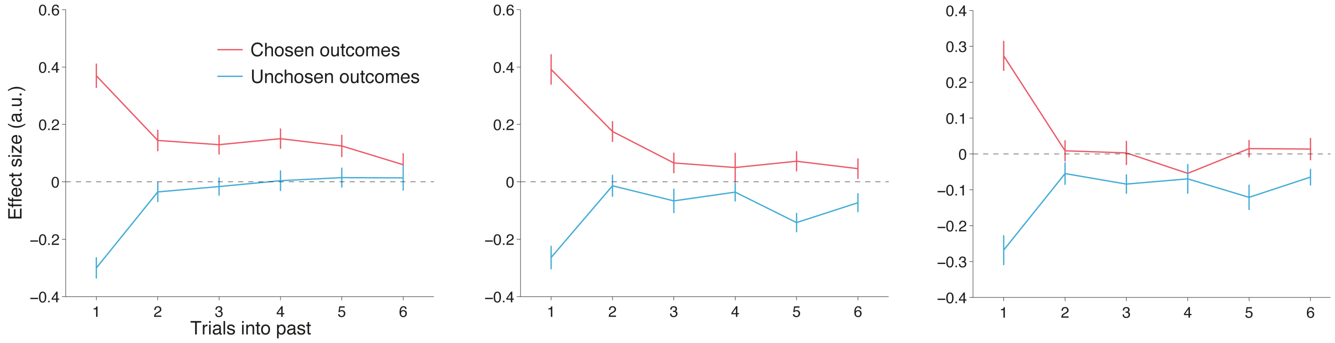

Supplementary Figure 1: Effects of chosen and unchosen outcomes on subjects' choices. Subjects' choices are guided by outcomes of both the chosen and unchosen option. Logistic regression results testing the effect of the outcomes on the chosen and unchosen option on the past six trials on subjects likelihood to stick with (positive regression coefficients) or switch away (negative coefficients) from the previous choice on the current trial. Left, middle and right panel show the results for experiments 1, 2 and 3, respectively. Values are mean ± SEM regression coefficients across subjects. Summing the effects over the past six trials, in experiment 1, the positive effect of chosen outcomes was bigger than the negative effect of unchosen outcomes (t_{29} = 3.76, p = 0.0008). In contrast, there was no difference in experiment 2 (p > 0.17), while in experiment 3, unchosen outcomes had a stronger effect compared to chosen outcomes (t_{29} = 4.65, p = 0.00016, all p-values report one-sample t-test).

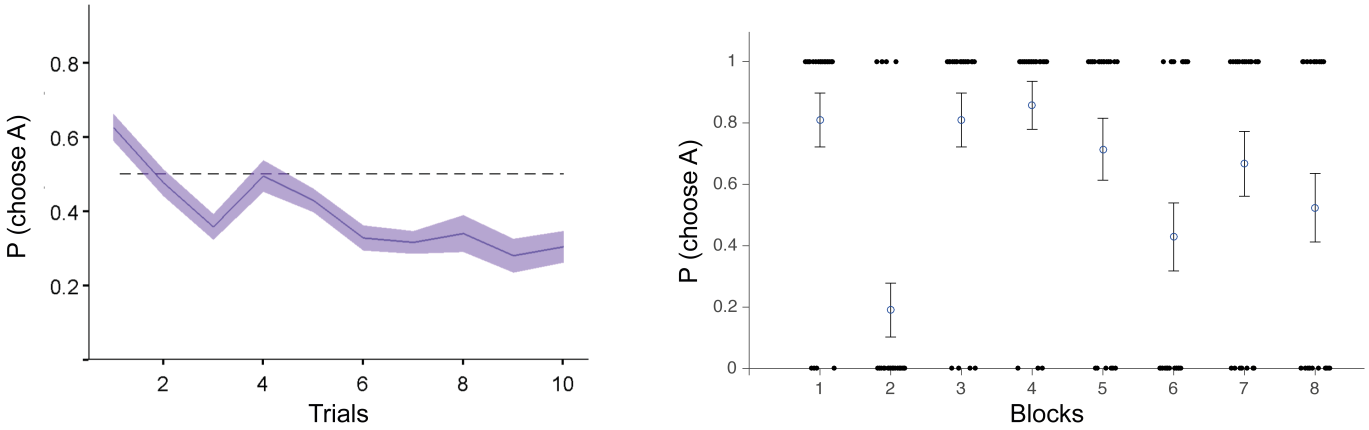

Supplementary Figure 2: Behaviour in the transfer phase of experiment 3. Both panels show the mean (across subjects) probability to prefer the objectively worse option A against C. Left: Choice behaviour on the ten transfer trials. Subjects' choices on the first transfer trial are biased towards the worse option A by virtue of previous learning experience, but new learning rapidly reverses this preference. Right: Choice behaviour for the very first transfer trial, shown separately for each of the eight blocks. Blue circles = mean, errorbars = SEM across subjects. Black dots represent individual data points.

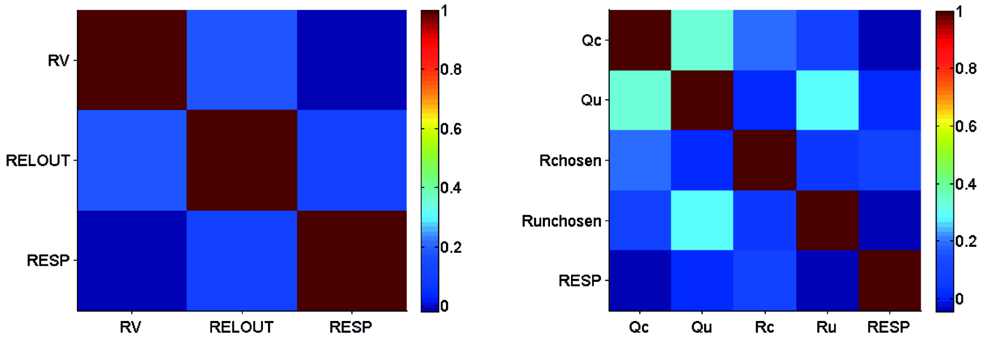

Supplementary Figure 3: Design matrix correlations. Average (across subjects) correlations between fMRI regressors in the design matrix for GLM 1 and GLM 2. RV = relative value, RELOUT = relative outcome, RESP = response, Qc = chosen value, Qu = unchosen value, Rchosen = chosen outcome, Runchosen = unchosen outcome.

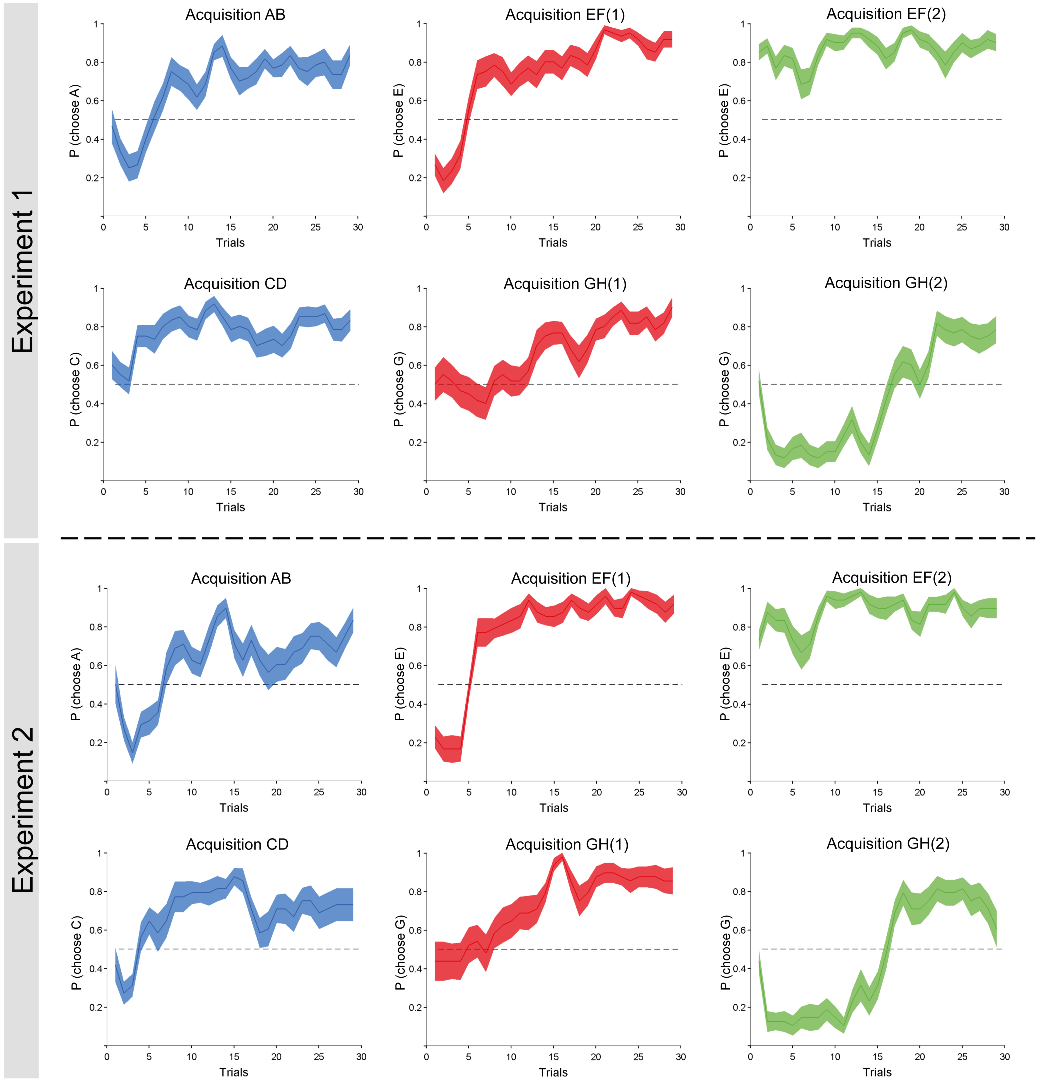

Supplementary Figure 4: Performance during acquisition of individual option pairs. Learning performance on pairs AB, CD, EF and GH. There were two rounds of EF and GH (with different stimuli, see main text), indicated by the number in parentheses. Values show mean ± SEM choice probability across subjects.

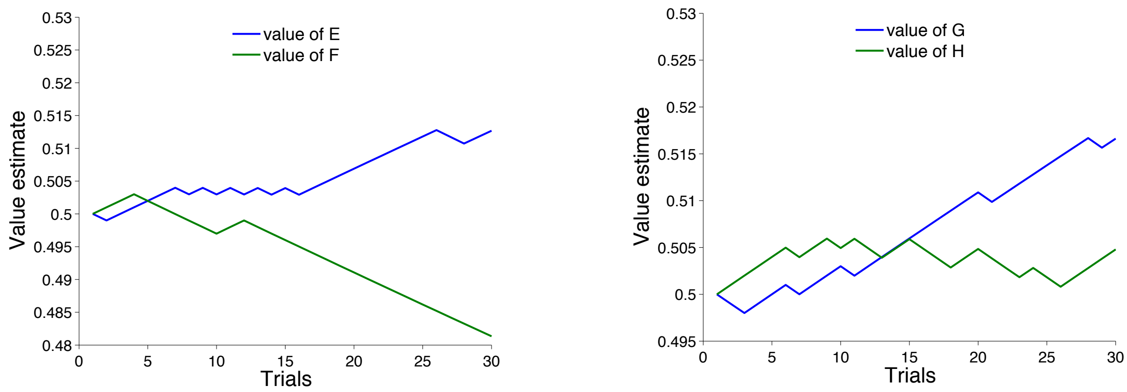

Supplementary Figure 5: Reinforcement learner value estimates for EF(1) and GH(2) learning. Supplementary figure 4 (above) shows below-chance performance on the initial trials of EF(1) and GH(2) discriminations. This is the result of a subtle initial advantage in local outcome histories for F over E, and H over G. The figure shows value estimates from a reinforcement learner, which reveals that the agent's estimate for H is higher compared to G during the initial ~15 trials, and higher for F compared to E during the first ~5 trials. The outcome sequences that participants experienced were:

Option E: 0 1 1 1 1 1 0 1 0 1 0 1 0 1 0 1 1 1 1 1 1 1 1 1 1 0 0 1 1 0
Option F: 1 1 1 0 0 0 0 0 0 1 1 0 0 0 0 0 0 0 0 0 0 0 0 0 0 0 0 0 0 1
Option G: 0 0 1 1 1 0 1 1 1 0 1 1 1 1 1 1 1 1 1 0 1 1 1 1 1 1 1 0 1 1
Option H: 1 1 1 1 1 0 1 1 0 1 0 0 1 1 0 0 0 1 1 0 0 0 1 0 0 1 1 1 1 1

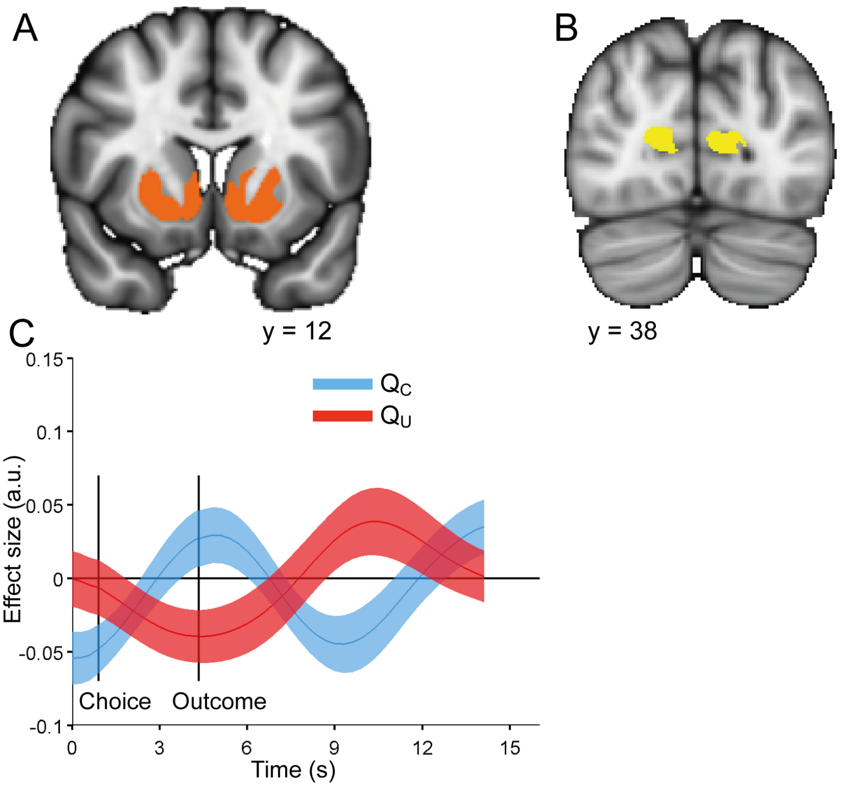

Supplementary Figure 6. A) Striatal region of interest used for the ROI analyses in main figure 4. B) Conjunction of positive responses to chosen and unchosen rewards at $p < 0.01$ reveals an area in bilateral primary visual cortex. C) Signal extracted from the occipital region in B (interpolated to a resolution of 300 ms) negatively encodes chosen value, Q_C.

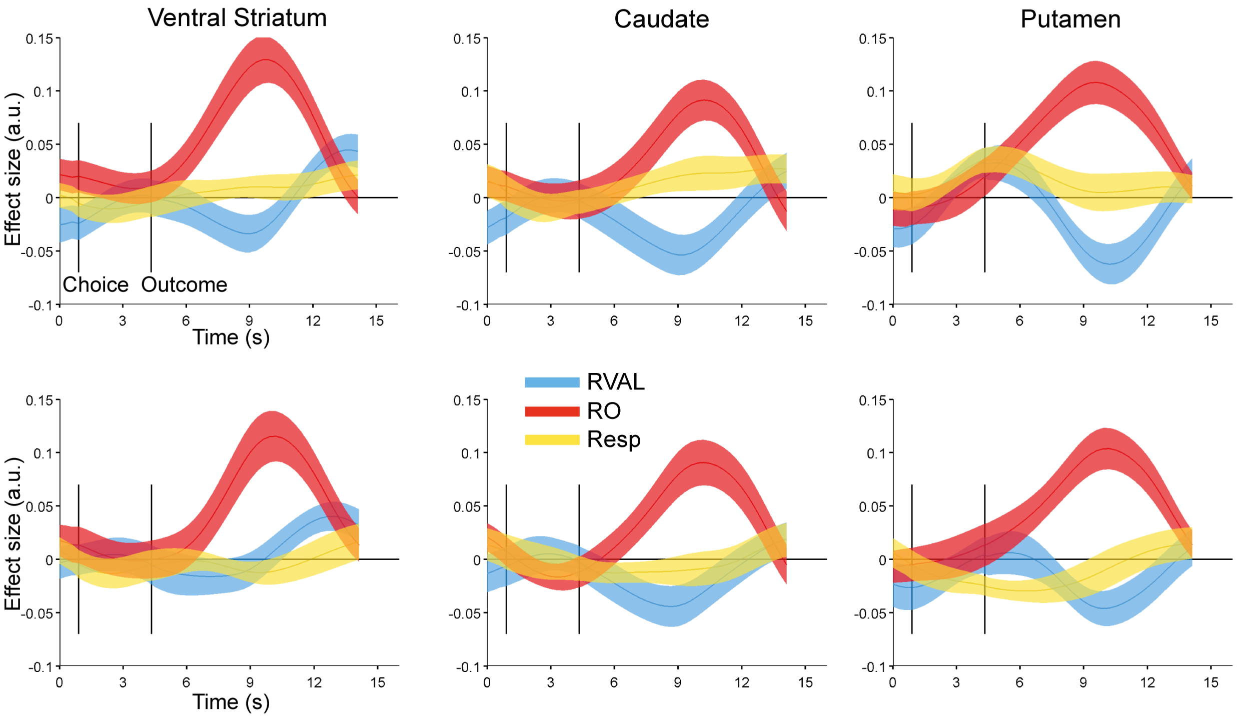

Supplementary Figure 7: Relative value and relative outcome coding in striatal subregions.
A negative effect of relative value was found both in caudate and putamen in both hemispheres (top row = left, bottom row = right hemisphere), whereas no such effect was found in the ventral striatum. The left ventral striatum did show an effect (peak t_{23} = 1.93), which however did not survive cluster-based correction. All regions showed a pronounced positive effect of relative outcome. Furthermore, qualitatively, representations of the motor response appeared to be more pronounced in the caudate and putamen compared to ventral striatum and to merge later in the caudate compared to the putamen. RVAL = relative value, RO = relative outcome, Resp = response made by the subject. BOLD signal is interpolated to a resolution of 300 ms.

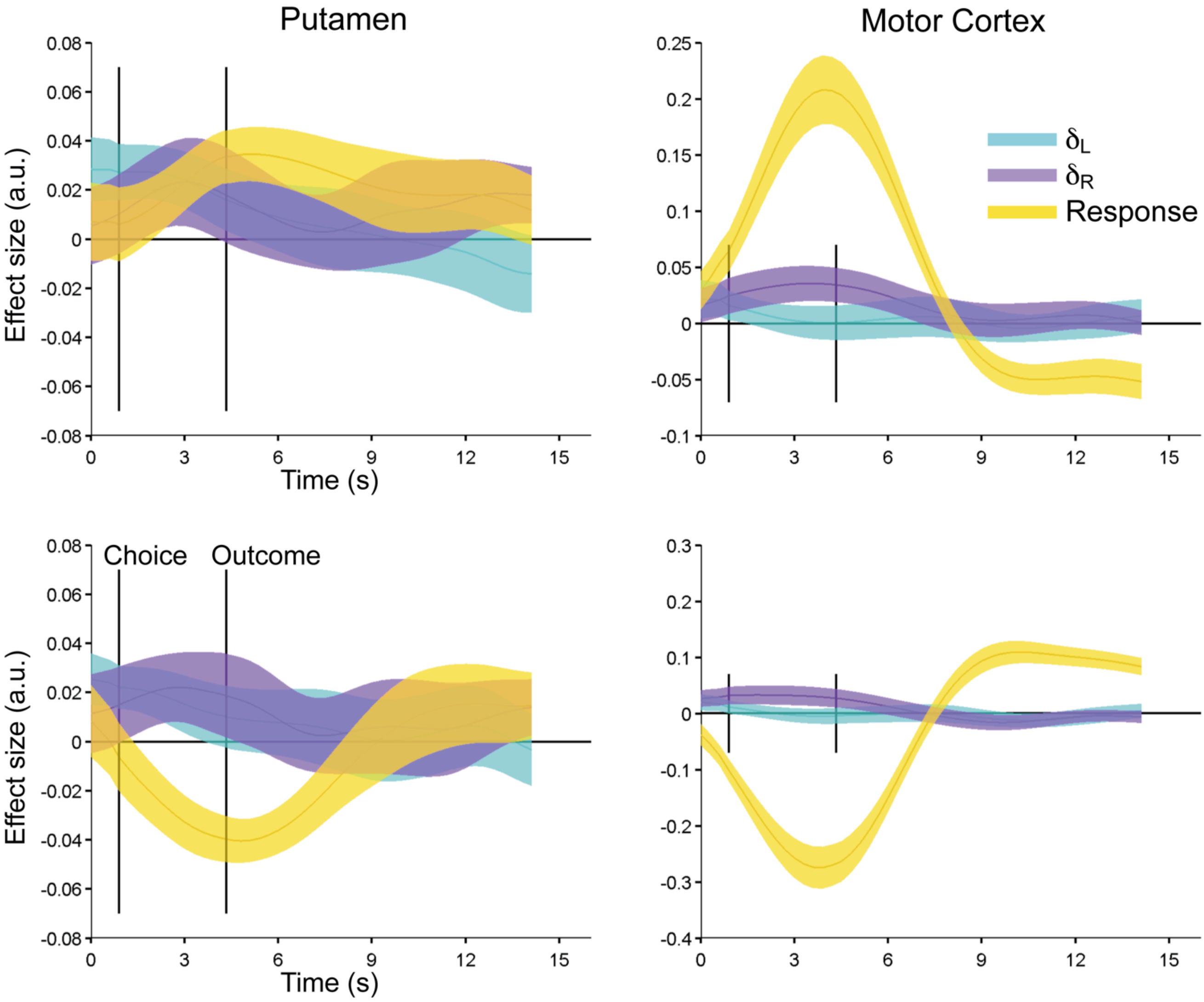

Supplementary Figure 8: Absence of action value prediction errors in putamen and motor cortex. Analysis investigating potential representations of action value prediction errors in motor corticostriatal circuitry. The effect of motor response (right vs left hand) is shown in yellow, prediction error effects for the left and right hand are shown in cyan and purple, respectively. The strong effect of response in motor cortex reflects a selection bias, since this ROI was identified based on a contrast of right minus left hand responses. Top row, left hemisphere, bottom row, right hemisphere. Despite pronounced coding of motor parameters, there is no evidence for a representation of action value prediction errors following outcome presentation in any of the four regions of interest. Solid lines represent mean, shaded areas SEM of regression coefficients across participants. BOLD signal is interpolated to a resolution of 300 ms.

Supplementary Table 1. Comparison of the relative value learner (RVL) used in the manuscript against different alternative learning mechanisms: A simple Q-Learner that updates both options with the same learning rate (Q-Learner), a Q-Learner with separate learning rates for the chosen and unchosen option (Q-C/U), a Q-Learner updating only the chosen option's value (Q-Learner chosen only), state-dependent relative value learning (SDL), which is the algorithm used by Palminteri and colleagues[1], and an actor-critic architecture (Actor-Critic). Upper panel gives model fits as negative log likelihood estimates (LL) and as Bayes Information Criterion (BIC), separately for both the entire acquisition (acq) and transfer phase (trans), and for the first transition trial (1st tra) in the four transitions (two type I and two type II transitions). Lower panel displays p-values for comparison of the RVL model against all other models tested here. In addition, for better intuition, the model negative LL on the first transfer trial is also exponentiated (in brackets) to give the models' average choice probabilities on the first transfer trial.

exp 1	LL acq	LL trans	LL 1st tra	BIC acq	BIC trans	BIC 1st tra
RVL	92.88	81.46	1.92 (0.62)	196.14	172.50	6.62
Q-Learner	92.88	85.79	3.34 (0.43)	196.14	181.15	9.47
Q-Learner chosen only	91.15	104.08	3.98 (0.37)	192.68	217.73	10.73
Q-C/U	88.82	82.50	3.38 (0.43)	193.22	180.58	10.92
SD RVL	70.70	67.57	3.07 (0.46)	162.16	154.29	11.68
Actor-Critic	88.33	83.41	3.04 (0.47)	192.24	181.19	10.23
exp 2						
RVL	93.96	80.84	1.88 (0.63)	198.30	171.26	6.53
Q-Learner	89.68	89.70	3.48 (0.42)	189.74	188.98	9.74
Q-Learner chosen only	93.96	82.40	3.43 (0.42)	198.30	174.37	9.63
Q-C/U	91.96	83.23	3.2 (0.45)	199.50	182.03	10.55
SD RVL	74.19	70.16	3.32 (0.44)	169.14	159.48	12.19
Actor-Critic	93.54	84.27	2.39 (0.55)	202.66	182.89	8.93

exp1			
RVL vs Q	p = 0.87	p = 0.086	p = 0.000024
RVL vs Q chosen only	p = 0.53	p = 0.0077	p = 0.0001
RVL vs Q c/u	p = 0.12	p = 0.229	p < 0.000002
RVL vs SD RVL	p = 0.0016	p = 0.021	p < 0.000002
RVL vs Actor-Critic	p = 0.013	p = 0.54	p < 0.000009
exp2			
RVL vs Q	p = 0.26	p = 0.0765	p = 0.0001
RVL vs Q chosen only	p = 0.35	p = 0.052	p = 0.0001
RVL vs Q c/u	p = 1	p = 0.052	p < 0.00002
RVL vs SD RVL	p = 0.092	p = 0.145	p < 0.00002
RVL vs Actor-Critic	p = 0.0012	p = 0.63	p = 0.0003

Supplementary Table 2. Estimated model parameters (median across subjects) for the six models described in the supplementary modelling below. Q-learner C = Q-Learner updating the chosen option only, Q-Learner C/U = Q-Learner updating both options but with separate learning rates, RVL = Relative Value Learner, SD-RVL = state-dependent Relative Value learning as recently used by[1]. α = learning rate, τ = softmax temperature, α_C and α_U = learning rates for the chosen and unchosen option, α_{State} = learning rate for estimating the average state value, α_{AC} and α_{CR} = actor and critic learning rates.

Q-Learner	α	τ
Exp 1	0.0015	0.01
Exp 2	0.039	0.058

RVL	α	τ
Exp 1	0.0014	0.02
Exp 2	0.039	0.11

Q-Learner C	α	τ
Exp 1	0.01	0.018
Exp 2	0.158	0.128

Q-Learner C/U	α_C	α_U	τ
Exp 1	0.044	0.01	0.062
Exp 2	0.051	0.012	0.083

SD RVL	α_C	α_U	α_{State}	τ
Exp 1	0.075	0.01	0.0007	0.08
Exp 2	0.072	0.016	0.0004	0.09

Actor-Critic	α_{AC}	α_{CR}	τ
Exp 1	0.037	0.112	0.095
Exp 2	0.017	0.1	0.036

Supplementary Table 3. Correlations between regressors in the design matrix used for the whole-brain analysis shown in figures 5 and 7A. Rc, Ru = chosen and unchosen outcome; Stim, Resp L, Resp R and outcome = main effects of stimulus presentation, L and R response, and outcome presentation, respectively.

	Rc	Ru	Stim	Resp L	Resp R	Outcome
Rc	1.00	0.22	-0.29	-0.14	-0.08	0.48
Ru	0.22	1.00	-0.25	-0.09	-0.11	0.42
Stim	-0.29	-0.25	1.00	0.33	0.33	-0.62
Resp L	-0.15	-0.09	0.33	1.00	-0.69	-0.24
Resp R	-0.08	-0.11	0.33	-0.69	1.00	-0.24
Outcome	0.48	0.42	-0.62	-0.24	-0.24	1.00

SUPPLEMENTARY METHODS

Computational modelling

Here, we describe the implementation of the two main models (absolute and relative learner) and five alternative models that we tested.

(1) Q-Learner with update of the chosen and unchosen option

This is a simple Q-Learner that estimates the objective reward probabilities using a simple Rescorla-Wagner update rule:

$$Q_{t+1} = Q_t + \alpha\delta_t \qquad [1]$$

Where Q_t is the estimated value on trial t, α is the subject specific learning rate and δ_t is the prediction error on trial t:

$$\delta_t = r_t - Q_t \qquad [2]$$

Where r_t is the reward (0 or 1) observed on trial t. The value estimates were then used to generate a probability for the model to select a given option (here: A vs B) using a softmax choice rule:

$$p_A = \frac{1}{1+ e^{-VD/\tau}} \qquad [3]$$

Where VD is the value difference between options (here: A and B) and τ is the softmax temperature that accounts for the stochasticity in subjects' choices.

(2) Relative Value Learner

This algorithm does not track separate value estimates for the two options in each pair. Instead, it directly learns how much better one option is compared to the alternative with which it is presented. It uses the same update rule as in equation [1]:

$$RV_{t+1} = RV_t + \alpha\delta_t \qquad [4]$$

However, here the prediction error δ_t takes the following form:

$$\delta_t = [Rc_t - Ru_t] - RV_t \qquad [5]$$

Where Rc_t and Ru_t are the rewards observed on the chosen and unchosen options, respectively. Thus, the outcome difference is compared to the expected outcome difference to update the relative value of options. Model choice probabilities were again given by a softmax function as in equation [3]

(3) Q-Learner with update of chosen option only

This agent is identical to model (1), with the only exception that it exclusively learns from direct experience, not using the outcomes on the non-chosen option to update the unchosen option's value. It thus captures the behaviour of a subject attending exclusively to the outcomes of the chosen option.

(4) Q-Learner with separate learning rates for chosen and unchosen options

Somewhere between the extremes of an agent learning exactly to the same extent from chosen and unchosen outcomes (model (1)) and an agent learning nothing at all from unchosen outcomes is an agent that learns from both, but to a greater or lesser degree from unchosen vs chosen outcomes. We capture this with a Q-Learner endowed with separate learning rates for the chosen and unchosen option. Again, values are updated using a simple delta rule:

$$Q_{t+1}(c) = Q_t(c) + \alpha_C \delta_t(c) \quad [6]$$

$$Q_{t+1}(u) = Q_t(u) + \alpha_U \delta_t(u)$$

Where α_C and α_U are the learning rates for the chosen and unchosen option, respectively.

(5) State-dependent relative value learning

Here, we describe the relative value learner as recently used by Palminteri and colleagues[1] that we used for comparison with our model. While the algorithm we used does not learn separate option values but instead directly learns how good one option is compared to the available alternative, the state-dependent relative value learner does learn separate option values. However, these are learnt with reference to the average value of the current context, or state. Option values are updated according to the same standard delta rule as in equation [1]:

$$Q_{t+1} = Q_t + \alpha \delta_t$$

With $\alpha = \alpha_C$ for the chosen option and $\alpha = \alpha_U$ for the unchosen option. However, here the prediction error δ_t on trial t for the chosen and non-chosen option is:

$$\delta_{C,t} = r_{C,t} - V(s)_t - Q_{C,t} \quad [7]$$

$$\delta_{U,t} = r_{U,t} - V(s)_t - Q_{U,t}$$

Where $V(s)_t$ is the state value on trial t, which is likewise updated on each trial:

$$V(s)_{t+1} = V(s)_t + \alpha_{State} \delta(s)_t \quad [8]$$

Where α_{State} is the state learning rate and $\delta(s)_t$ is the state prediction error on trial t:

$$\delta(s)_t = SO_t - V(s)_t \quad [9]$$

Where the state-level average outcome SO is represented by the average reward on the chosen and unchosen option:

$$SO_t = \frac{1}{2}(r_{C,t} + r_{U,t}) \qquad [10]$$

This algorithm is strongly related to Baird's advantage updating, from which it differs in terms of the inclusion of counterfactual learning and by comparing the selected action with the average outcome, rather than the best outcome[2].

(6) Actor-Critic learning

Actor-Critic learning has in common with state-dependent relative value learning (model (5)) the learning of a state value function and prediction errors that are based on this state value estimate. The actor selects an action based on a policy π and this action is evaluated by the critic. Unlike in other forms of learning, there is a separate representation of the policy, independent of the value function. On each trial, the action selected by the subject generates a prediction error δ_t:

$$\delta_t = r_t - V(state)_t \qquad [11]$$

Where $V(state)_t$ is the value of a particular state, where pairs of stimuli presented together represent one state. The resulting prediction error is then used to update both the state value (the critic) and a separate policy (the actor):

$$V(state)_{t+1} = V(state)_t + \alpha_C \delta_t \qquad [12]$$

$$\pi(s,a)_{t+1} = \pi(s,a)_t + \alpha_A \alpha \delta_t \qquad [13]$$

Where the policy $\pi(s, a)$ is the strength of the connection between the chosen stimulus and the action of selecting it when in state s, and α_A and α_C are the learning rates in the actor and the critic, respectively. Policy weights are then again used for action selection using a softmax rule:

$$p(s,a1)_t = \frac{1}{1+e^{-[\pi(s,a1)_t \; - \pi(s,a2)_t]/\tau}} \qquad [14]$$

The free parameters in all models were estimated using custom-written model fitting procedures in Matlab. The parameter space was set up as n-dimensional grids in log space (where n is the number of parameters in the respective model). Negative log likelihoods were computed for each parameter combination in the grid:

$$-LLE = -\sum_{i=1}^{t} \log (p_t) \qquad [15]$$

Where p_t is the model's choice probability on trial t. The grid optimum was then used to initialise further optimisation using the Nelder-Mead simplex algorithm implemented in Matlab's fminsearch function.

ROI analyses

The BOLD timeseries from regions of interest were resampled to a resolution of 300 ms using cubic spline interpolation before being cut into trials with a duration of 14.4 s. Each trial consisted of three phases: CHOICE (time between stimulus and response onset), DELAY (time between response and outcome onset), and OUTCOME (a fixed window of 10 s following outcome onset). The duration of both DELAY and OUTCOME were fixed (3.5 and 10 s), whereas the CHOICE phase was of variable duration, depending on subject's response time (RT) on a particular trial. As duration for the CHOICE phase, we used 0.9 s, corresponding to the mean RT across trials and subjects. Thus, the BOLD signal was cut into epochs of 14.4 s on each trial (0.9 s CHOICE, 3.5 s DELAY, and 10 s OUTCOME), where the start of each phase was defined by the exact onset of each event in each trial. The variability of the RT means that on trials with faster than average RT, the last few data points at the end of the CHOICE phase contain data points that actually belong to the first samples of the DELAY phase. Conversely, on trials with longer than average RT, the last few data points of the CHOICE phase will be missing from the analysis. In the plots, subtle discontinuities at the transition between the CHOICE and DELAY phase are the result of this. Note that all of our analyses exclusively focus on the OUCOME period, which is of a constant duration and thus is not affected by this procedure. The resulting data matrix is of size m x n, where m = number of trials and n = number of timepoints. We then regressed a design matrix X against this data matrix at each time poing using ordinary least squares regression. The design matrix X is of size m x p, where p = number of regressors. This results in a p x n matrix, which is the timecourse (n time points) of regression coefficients for each regressor p.

SUPPLEMENTARY REFERENCES

1 Palminteri, S., Khamassi, M., Joffily, M. & Coricelli, G. Contextual modulation of value signals in reward and punishment learning. *Nat Commun* **6**, 8096, (2015).

2 Baird, L. C. *Advantage updating*. (Technical Report WL- TR-93-1146, Wright-Patterson Air Force Base, 1993).
