# Supplementary material for: Publisher Correction: Learning relative values in the striatum induces violations of normative decision making
Source: Nat Commun. 2019 Jun 27;10:2833. doi: 10.1038/s41467-019-10718-8 (PMC6597713; doi:10.1038/s41467-019-10718-8)
Supplement: Supplementary file 1 — Supplementary Figures [file 41467_2019_10718_MOESM1_ESM.zip › readme.pdf]

This folder contains the behavioural data for all three experiments reported in the paper "Learning relative values in the striatum induces violations of normative decision making", Klein et al., 2017.

Exp 1 and Exp 2

Exp1 and exp2 contain the data for the behavioural and fMRI study, respectively. Exp3 contains the data from the third (behavioural) study that is shown in figure 3.

The data format is as follows for experiments 1 and 2:
There is one file per subject in each folder. Each file consists of a text header (to be ignored) and data. The data is organised as trials in rows and parameters in columns, with the following columns:

1: stimulus presented on the left (number 1-12 for stimuli A, B, C, D, E1, F1, G1, H1, E2, F2, G2, H2)
2: stimulus presented on the right (coding as above for column 1)
3: reward available on left option or not (1/0)
4: reward available on right option or not (1/0)
5: choice made by subject (1 = left, 2 = right, NaN = missed response)
6: onset of stimulus (in ms)
7: onset of response (in ms)
8: onset of outcome (in ms)
9: RT (in ms)
10: Trial type (1=AB, 2=CD, 3=AC, 4=BD, 5=EF1, 6=GH1, 7=E1G1, 8=EF2, 9=GH2, 10=E2G2)
11: Experiment phase (1 = acquisition, 2 = transfer)

Overall, there are 180 trials for acquisition and 120 trials for transfer. When a subject failed to respond within the required time, this trial was repeated. Some data sets may therefore have more than 300 rows. The data with response misses have to be excluded for analysis.

Exp 3

The data for experiment 3 is organised the same way as for experiments 1 and 2, with the following exceptions:

1) Columns 10 and 11 are to be ignored (they just two columnns of ones throughout)
2) Columns 1 and 2 still code for the stimulus that was presented on the left and right, but numbered 1 – 32.

There are eight blocks. In each block, each pair of AB and CD trials is presented 40 times altogether in random order, so there are 80 trials of acquisition altogether. This is followed by 10 transfer trials, in which AC trials are presented. In each new block, new symbols are used for the four options, represented by the number in columns 1 and 2, eg. [1,2,3,4], [5, 6, 7, 8], ..., [29, 30, 31, 32]. The assignment of symbol numbers to options always is in ascending order to A, B, C, D – e.g. for the second block, this would be A = 5, B = 6, C = 7, D = 8.

There are 8 blocks x 90 trials = 720 trials in total.
